# The Brain Signature of Reward Processing During Cooperative Gaming

**DOI:** 10.1111/psyp.70341

**Published:** 2026-07-28

**Authors:** Karl‐Philipp Flösch, Tobias Flaisch, Marco Steinhauser, Harald T. Schupp

**Affiliations:** ^1^ Department of Psychology University of Konstanz Konstanz Germany; ^2^ Centre for the Advanced Study of Collective Behaviour University of Konstanz Konstanz Germany; ^3^ Department of Psychology Catholic University of Eichstätt‐Ingolstadt Eichstätt Germany

**Keywords:** brain oscillations, cooperation, ERP, experimental game, hyperscanning, reward

## Abstract

Human cooperation depends on shared goals and dynamic role‐taking, aligning individual actions with goal‐related tasks. However, in everyday social interactions, cooperation is often intertwined with personal goals and motivations. In this study, 48 young, healthy participants played the dyadic Pacman Game that incorporated a personal gamble to reflect these social dynamics. Brain oscillations and event‐related potentials differentiated between achieving a shared goal enhanced by personal rewards and reaching a shared goal without a personal reward. Specifically, personal rewards, which had to be inferred from social cues rather than explicit feedback, were linked to increased delta/theta power and sustained positive ERP potentials over fronto‐central regions. Furthermore, we replicated findings of alpha/beta power decreases and enhanced P3‐like positivities associated with cognitive and semantic processing demands of specific player roles. Our results highlight how neural measures obtained in experimental games shed light on the interplay between shared goals and personal motivations, offering a valuable framework to bridge insights from controlled paradigms to real‐world social cooperation.

## Impact Statement

Cooperation in daily life involves both shared goals and personal motivations. Using a role‐based dyadic Pacman Game with EEG hyperscanning, we show that personal rewards inferred from social cues shape neural responses during cooperation, while established markers of role‐specific task demands are replicated. These findings highlight naturalistic experimental games as a powerful bridge between controlled paradigms and real‐world social interaction.

## 1 Introduction

Human culture, in its diverse forms, is fundamentally rooted in the capacity for social cooperation (Henrich and Henrich 2007; Tomasello 2019). This capacity relies on a core motivation to engage in social interaction (Tomasello et al. 2005) and on complex cognitive processes that enable individuals to coordinate their actions toward shared goals (Knoblich et al. 2011; Shultz and Dunbar 2012). To examine the cognitive and motivational mechanisms supporting such coordination, researchers frequently employ controlled experimental games that assign complementary roles to interaction partners, thereby enabling the systematic investigation of role‐specific processes. In the present study, we examined whether neural markers of reward processing are sensitive to an additional personal incentive embedded within an otherwise shared cooperative outcome.

Experimental games provide a valuable tool for examining social cognitive processes underpinning cooperation. By establishing rule‐governed interaction contexts, in which specific actions carry defined meanings (Huizinga 1956; Perinbanayagam 2006), games allow researchers to systematically manipulate roles, goals, and motivations while maintaining ecological relevance. In social neuroscience, paradigms such as the Prisoner's Dilemma and trust or ultimatum games have been widely used to uncover neural mechanisms underlying strategic decision‐making and social evaluation (e.g., Jahng et al. 2017; King‐Casas et al. 2005; Rilling and Sanfey 2011; Tomlin et al. 2006). Moving toward more naturalistic contexts, recent approaches extended these paradigms to dynamic, turn‐based interactions, thereby enabling the investigation of temporally unfolding coordination processes. For example, “stag hunt” games have revealed increased activity in mentalizing networks during collaborative decisions (Yoshida et al. 2010), and dyadic word‐by‐word interactions have linked turn‐taking dynamics to modulations of the N400 and late positive^1^ (P600) event‐related potential (ERP) modulations (Goregliad Fjaellingsdal, Schwenke, Ruigendijk, et al. 2020; Goregliad Fjaellingsdal, Schwenke, Scherbaum, et al. 2020). Together, these findings highlight the value of experimental games for studying neural processes underlying social cooperation.

**FIGURE 1:**
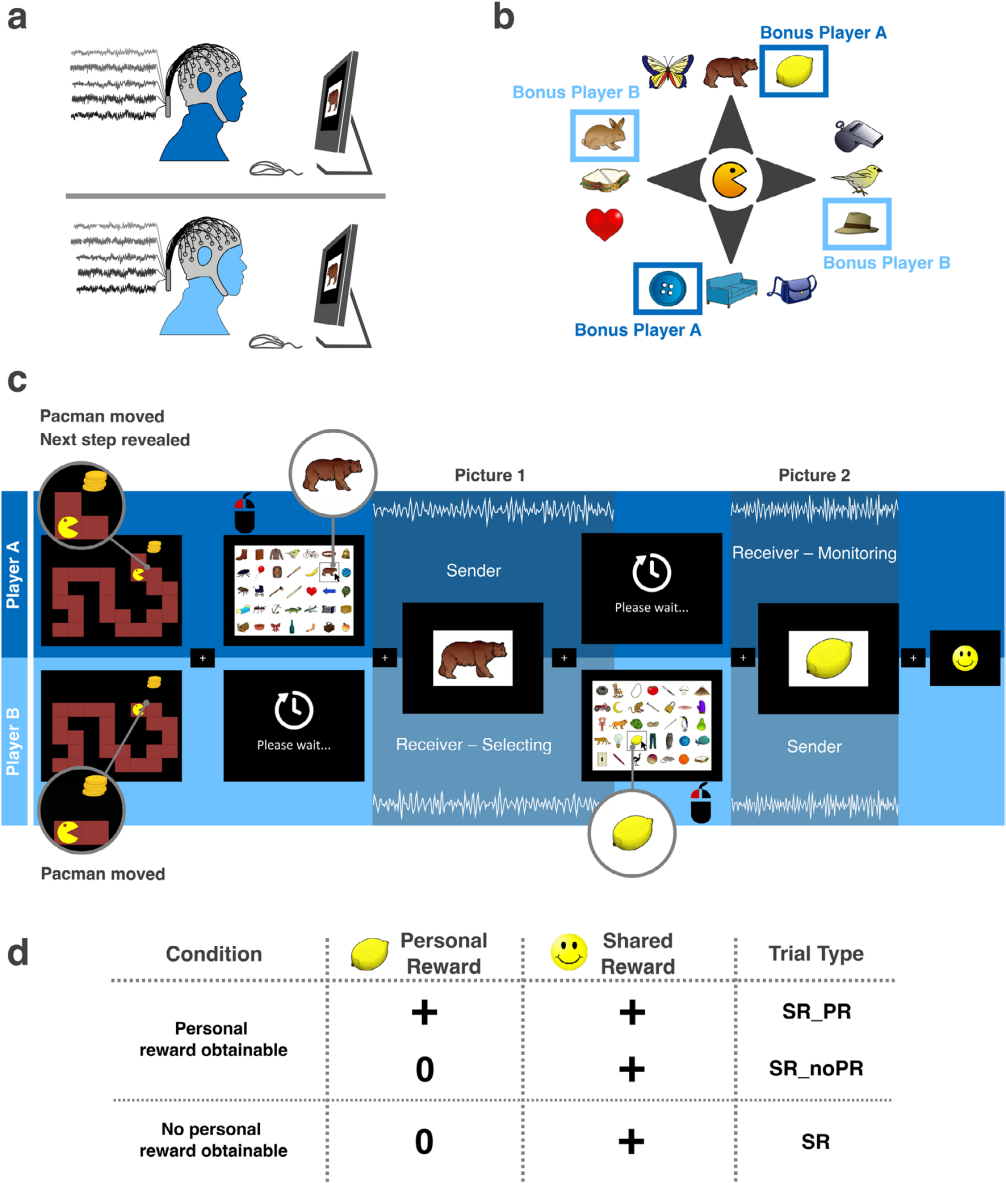
Schematic depiction of the Pacman Game. (a) Game setup: Participants play a dyadic game while 257 channel EEG is measured simultaneously from both players. The players communicate by sending symbolic picture cues (e.g., *bear* + *lemon* = *up*). (b) Coding scheme: Before starting the game, players agree upon a symbolic coding scheme associating three picture cues with each of the four moving directions. For each player, two cues from different directions are randomly selected to indicate the reception of a personal reward. (c) Experimental trial: In each game trial, one player is informed about the next moving direction by uncovering the subsequent step in the maze. This informed player acts as the sender of Picture 1, selecting a picture cue to communicate the moving direction to the receiver, who awaits its presentation. Upon receiving Picture 1, the receiver selects the complementary picture cue (e.g., the lemon), thereby becoming the sender of *Picture 2*. Feedback follows: a smiley face indicates the correct selection of cues and a shared reward (*SR*), while a frowning face signals an error. Additionally, depending on the second picture cue, the monitoring receiver can earn a personal reward (*PR*). At the start of the next trial, the maze reappears, showing the Pacman's movement to the next position, and game roles are randomly re‐assigned by revealing the moving direction to one player. The present example shows a personal reward trial for Player A. The correct moving direction (*up*) is initially revealed to Player A who accordingly acts as sender of Picture 1 (*bear*). Player B, as the selecting receiver, then chooses *Picture 2* (*lemon*), signaling to the monitoring receiver (Player A) that a personal reward is obtained (*SR_PR* trial; see panel b and d). Please note that screens depicted at the middle line were presented to both players identically. All screens had the same dimensions, size differences are for illustrative purposes. The mouse pictogram indicates a response by the respective player. The schematic EEG signal denotes the epochs selected for data analysis. (d) Personal reward: The three trial types used to analyze reward processing effects. This figure is based on Flösch et al. (2024a) and adapted for the present paradigm.

Shared goals and the implicit agreement to pursue them provide strong motivation, aligning individual actions with goal‐related tasks structured by collaborative roles (Gallotti and Frith 2013; Levinson 2006; Searle 1995; Tomasello 2019). Two recent studies employed the ‘Pacman Game’ (see Figure 1) to investigate social role taking between two interaction partners (Flösch et al. 2024b, 2024a). Players cooperatively navigated a Pacman through a maze using an agreed‐upon symbolic coding scheme. In each move, they completed two consecutive turns, alternating roles as sender and receiver of picture cues. The cues differed in informational value depending on the current role, with receivers facing higher cognitive demands than senders. Accordingly, receivers exhibited enhanced early negativities (~160–210 ms) and P3 amplitudes (~300–600 ms), as well as sustained decreases in alpha/beta oscillations (~400–2500 ms), relative to senders (Flösch et al. 2024b, 2024a). In addition, semantic processing demands varied between receiver turns, eliciting a distinct late positivity (~350–600 ms) and reduced alpha to lower beta power (~8–20 Hz; ~400–1400 ms). These findings provide initial evidence that specific ERP components and alpha/beta oscillations index cognitive brain states associated with role‐dependent demands during social cooperation in games.

Building on these findings, the present study extended the Pacman Game to incorporate motivational factors involved in gameplay. In everyday life, social situations are often complex, with cooperation intertwined with personal goals and motivations. Consequently, when individuals pursue common goals, the same outcome may carry greater significance for one collaborator than for another. For instance, if you are collaborating with colleagues on a project and your promotion depends on its success while theirs does not, the outcome will naturally hold more importance for you. Accordingly, we aimed to investigate whether established neural markers, that is, brain oscillations and event‐related potentials, could differentiate between achieving a shared goal and achieving a shared goal enriched by an additional personal reward.

Candidate measures for evaluating personal rewards were drawn from prior research on reward processing in individual‐focused experimental paradigms. Previous research using ERPs show that feedback indicating monetary reward, as opposed to neutral or loss feedback, elicits a fronto‐central Reward Positivity (RewP) from about 200–400 ms, thought to reflect reward prediction errors, appearing when outcomes are better or worse than expected (Proudfit 2015; Sambrook and Goslin 2015). Subsequent effects include broadly distributed P3 and late positive potential (LPP) components between ~300 and 1000 ms, presumed to reflect evaluative and affective aspects of reward processing (Donaldson et al. 2016; Foti et al. 2011; Glazer et al. 2018; Meadows et al. 2016; Yeung and Sanfey 2004). Another line of research has investigated how personal rewards affect oscillatory brain activity, with the theta and delta bands (~1–8 Hz) showing sensitivity to feedback indicating monetary reward (Cohen et al. 2009, 2007; HajiHosseini et al. 2012; Marco‐Pallarés et al. 2008; Williams et al. 2021). For instance, in a dual‐choice gambling task, Cohen and colleagues observed increased delta to theta power during feedback over fronto‐central sensor sites (~300–600 ms), with the magnitude of these effects modulated by the reward prediction error, that is, inversely correlated with the probability of receiving a reward (Cohen et al. 2007, 2009). In sum, cognitive neuroscience has consistently demonstrated a link between ERP and oscillatory neural markers and the preferential processing of stimuli associated with personal reward.

The present study advances our research program by investigating affective and cognitive processes within a naturalistic social cooperation context. Collaborative tasks often involve personal goals and motivations, which can lead to differences in stimulus relevance among collaborators. To experimentally influence individual motivation, we incorporated a personal gamble into the Pacman Game. Specifically, certain directional cues were uniquely linked to a bonus reward for each participant (see Figure 1b). By design, the personal bonus cue could only appear for receivers during the second turn of a correct move (see Figure 1c), thereby augmenting the shared reward associated with successful gameplay through an additional personal incentive (see Figure 1d). To identify neural markers of personal reward within a naturalistic interaction context, we analyzed event‐related potentials, reflecting phase‐locked neural activity, as well as induced brain oscillations capturing non‐phase‐locked dynamics. Importantly, in the present paradigm, personal reward was embedded within the task and had to be inferred from the identity of the directional cue rather than being conveyed through an explicit feedback signal. This design more closely resembles everyday social interactions, in which the personal significance of an outcome is often derived from contextual information rather than discrete feedback events. We therefore hypothesized that this implicit reward structure would engage neural mechanisms comparable to those observed in classical reinforcement‐learning paradigms. Specifically, cues signaling a personal reward were predicted to (1) increase delta‐to‐theta band power over fronto‐central regions and (2) enhance RewP, P3, and LPP amplitudes.

Furthermore, continuing our efforts to ensure replication, we aimed to replicate all findings reported in our previous research (Flösch et al. 2024a, 2024b). Accordingly, we expected that the sender‐receiver asymmetry, driven by the cognitive demands placed on the receiver, would be associated with larger amplitudes in specific ERP components (early negativity, P3, and late positivity) as well as an event‐related desynchronization (ERD) in the alpha to lower beta band.

## 2 Method

### 2.1 Participants

Forty‐eight healthy students (30 female; two left‐handed) were recruited on‐campus at the University of Konstanz and participated in dyads. To identify brain states related to reward processing and to replicate previous findings (Flösch et al. 2024a, 2024b), sample size determination was conducted for ERP components and oscillatory brain activity. For the analysis of brain oscillations, a power simulation using the FieldTrip cluster permutation test was computed (Wang and Zhang 2021). Based on a paired mean difference of 1.0 dB, a standard deviation of 1.6 dB, and 1000 cluster permutations per iteration, a sample size of 45 participants was estimated to detect effects at *p* = 0.001 with 90% statistical power. Regarding ERP analyses, prior findings were based on sample sizes ranging from *N* = 24 to 32 with up to 52 trials per condition. Since the critical experimental condition in the present study, targeting personal reward effects, yielded a maximum of only 26 trials and in order to counterbalance experimental cells, the sample size was increased by a factor of 1.5. Overall, a sample of *N* = 48 participants was examined to assess ERP and oscillatory effects.

Participants were between 18 and 33 years of age (*M* = 22.6, SD = 3.1) and reported no neurological or psychiatric disorders. They had normal or corrected‐to‐normal vision. They received a fixed monetary compensation of 20€ or course credits for their participation, with additional monetary reward dependent on task performance. The study protocol was approved by the Institutional Review Board of the University of Konstanz in accordance with the regulations of the Declaration of Helsinki. All participants provided written informed consent and were debriefed after the experimental session.

### 2.2 Stimulus Material

256 colored pictures were taken from Snodgrass and Vanderwart's object pictorial set (Rossion and Pourtois 2004; Snodgrass and Vanderwart 1980). Each picture was presented either as original or as a mirrored duplicate to avoid the use of directional cues (e.g., “The fish swims from right to left”). Stimuli were presented on a 27‐in. LED monitor (60 Hz refresh rate) located ~105 cm in front of the participants, resulting in horizontal and vertical visual angles of about 14° × 14°.

### 2.3 Experimental Task

Participants played the Pacman Game (Flösch et al. 2024a), which was enriched by the possibility of obtaining personal rewards during gameplay.

Preceding the main experiment, the two participants engaged in a brief social ice‐breaking activity by playing the word guessing game “Taboo” to familiarize themselves with each other.

Subsequently, they were individually seated in separate EEG chambers. To promote a collaborative game environment, participants were connected through a live video stream (Zoom Video Communication, CA) using additional notebooks with integrated webcams positioned to the right of each participant.

#### 2.3.1 The Pacman Game

As illustrated in Figure 1, participants were instructed to collaboratively navigate a Pacman figure from start to goal. Instead of actual directional cues, they developed a unique symbolic code for up, down, left, and right movements which was used during the entire experiment. Through a video stream, the game partners agreed on this code before starting the game, selecting three pictures per direction from a random subset of 32 unique images. They were encouraged to create a story or rule of thumb to help memorize the 12 pictures and their meanings.

Each game began with a stack of golden coins marking the goal position. The concealed maze path consisting of 26 steps was revealed incrementally with each trial. Both players saw the Pacman's starting position for 3000 ms, but only one player viewed the next step, thereby establishing the players' roles for the upcoming trial. The informed player acted as the sender initially and then as the monitoring receiver (e.g., *Player A*; see Figure 1c), while the uninformed player acted as the selecting receiver first and then as the sender (e.g., *Player B*). After presentation of a fixation cross (500 ms) the first selection array appeared, giving the informed player (*Player A*) 6000 ms to choose the picture indicating the correct moving direction. The selection array included one of the three symbolic cues per direction, along with distractor cues, resulting in a total of 35 cues (see Figure 1c). The first response terminated the presentation of the selection array. Meanwhile, the uninformed player saw a screen prompting them to wait for the partner's response. Following this, a fixation cross (500 ms) followed by the selected picture cue (1000 ms; Picture 1) were shown to both players, that is, the sender and the selecting receiver. Another fixation cross (500 ms) followed. In the second selection array (6000 ms time limit), the player roles switched, with *Player B* now acting as the sender and selecting a complementary picture cue (Picture 2) corresponding to *Player A*'s indicated direction. The second selection array always showed a different symbolic cue than the first array for the correct direction. After a fixation cross (500 ms) the second picture cue (Picture 2) was displayed for 1000 ms to both players, that is, the sender and the monitoring receiver. After a fixation cross (500 ms), feedback (1500 ms) was provided via a smiling face for correct responses or a frowning face for incorrect or delayed responses, with each correct trial yielding a shared reward of 1€. The next game move then started with a display of the Pacman's step shown to both players (3000 ms) along with the moving direction information revealed only to one player.

Player roles were re‐assigned each trial and were indicated by uncovering or not uncovering the next maze step. Random assignment ensured that each player took the different roles equally often over each game. Each game had 26 steps, with participants playing eight games, totaling 208 trials. Each of the four moving directions appeared equally often (i.e., 52 times) across the eight games. To maintain the predetermined 26‐step pathway through the maze, the Pacman figure moved to the next location irrespective of the players' responses.

#### 2.3.2 Personal Reward Trials

Each player was presented with two picture cues designated as their “personal bonus symbols”, which they were required to memorize. They were informed that each time they received one of these pictures as a symbolic cue during playing, they would earn an additional 2€ as a personal reward. Each set of four bonus cues covered all directions, with each player getting two symbols that indicated two different directions (see Figure 1b). The reward cues were reassigned before each game, and their use was balanced across the four directions, the two players, and the eight games. Thus, 32 cue assignments occurred in total across the experiment, such that each of the 12 cues served as a reward cue on average 2.7 times, varying slightly across cues. Personal reward cues could only appear in the position of Picture 2 and only for the monitoring receiver. Throughout the experiment, in half of the game trials, the Pacman moved in a direction associated with one player's reward cues, while in the other half, it moved in a direction linked to the reward cues of the other player. In these trials, the monitoring receiver had the chance to obtain a personal reward, which was randomly granted in half of the cases (26 *SR_PR* trials) and withheld in the other half (26 *SR_noPR* trials). In the remaining game trials, the player never received a personal reward (156 trials; 52 *SR* trials as the receiver of Picture 2 + 104 *SR* trials as the sender of Picture 2).

### 2.4 Procedure

Each of the eight games lasted for about 10 min. During the game, communication relied solely on picture cues, with verbal communication precluded by muting the Zoom meeting. Participants were also instructed to focus on the center of the main screen and not to look at their game partner. During brief breaks between games, participants could relax and were encouraged to converse with their game partner.

At the end of the experiment, the rewards were determined by randomly selecting a game (by rolling dice) for both the shared and personal rewards, respectively. Participants earned on average 17.52€ (SD = 2.18), comprising half of the shared reward (*M* = 22.13€; SD = 2.42) and their personal bonus (*M* = 6.46€; SD = 1.81). Participants recalled their personal bonus cues with a mean accuracy of 93.4% (SD = 1.8).

### 2.5 Behavioral Performance

To assess effects of player roles and personal reward, we analyzed the behavioral performance of participants, that is, reaction times and error rates, by means of two‐way ANOVAs with repeated measurement on the factors of *Position* (Picture 1 vs. Picture 2) and *Trial Type* (SR vs. SR_noPR vs. SR_PR).

Regarding reaction time, players responded generally slower during the first visual search task (Picture 1*; M* = 2663 ms; SD_trials_ = 1150; SD_subjects_ = 278) than during the second task (Picture 2*; M* = 2446 ms; SD_trials_ = 1121; SD_subjects_ = 243), *F*(1,47) = 47.6, *p* < 0.001, $\eta_{g}^{2}$ = 0.11, $\eta_{p}^{2}$ = 0.50, which, however, was unaffected by whether the players could obtain a personal reward or not (all effects involving *Trial Type*: *Fs* ≤ 1.71, *ps* ≥ 0.191).

Regarding response accuracy, the proportion of errors was 10.4% (SD = 4.8) for the first and 9.8% (SD = 4.8) for the second visual search task. No effect reached significance (*Fs* ≤ 1.45, *ps* ≥ 0.239).

### 2.6 EEG Recordings and Statistical Analysis

#### 2.6.1 EEG Data Acquisition

Data acquisition was conducted using two 257‐channel geodesic sensor nets (GES 300 v2.0; EGI: Electrical Geodesics Inc., Eugene, OR), with data collected via Netstation acquisition software and two EGI DC‐amplifiers (Net Amps 300 & 400). The vertex electrode (Cz) served as an online reference, and electrode impedance was maintained below 30 kΩ, as per EGI guidelines for this EEG system. Data were recorded DC, sampled at 1000 Hz, and subjected to online lowpass filtering at the Nyquist frequency. Data processing involved MATLAB (The Mathworks Inc., Natick, MA), FieldTrip (Oostenveld et al. 2011), and EMEGS (Peyk et al. 2011) software.

Continuous EEG data were filtered offline using one‐pass zero‐phase hamming‐windowed sinc FIR filters. Specifically, a highpass filter with a half‐amplitude cutoff (−6 dB) at 0.1 Hz (order 16500, transition width of 0.2 Hz, passband from 0.2 to 500 Hz) and a lowpass filter with a half‐amplitude cutoff (−6 dB) at 120 Hz (order 110, transition width of 30.0 Hz, passband from 0 to 105 Hz, stopband from 135 to 500 Hz) were used, both with a maximal passband deviation of 0.0022 dB (0.22%) and a stopband attenuation of −53 dB. Epochs were extracted from 500 ms before to 1000 ms after stimulus onset for the two picture cues as well as the second selection array. Using an Independent Component Analysis (ICA) performed on concatenated epochs, an average of 17.1 (SD = 5.9) components related to ocular and muscle artifacts were rejected. Channels and epochs with high variance, indicative of movement artifacts, were excluded based on visual inspection. Data were then converted to an average reference and DFT‐filtered to remove line noise at 50 and 100 Hz. Removed channels (*M* = 10.9, SD = 2.2) were interpolated using an unweighted average of neighboring sensors, and *M* = 82.9% (SD = 7.2) of epochs were retained. In order to assure that a shared reward was received in all three trial types, only epochs from correct trials were used. The proportion of epochs used did not vary significantly across experimental conditions, χ^2^(9) = 0.90, *p* = 0.99.

Computation and statistical comparisons of power spectra as well as cluster‐based permutation testing were conducted using FieldTrip. Statistical analyses of oscillatory and ERP data were calculated using R software (R Core Team 2022) including the R‐packages “tidyverse” (Wickham et al. 2019), “afex” (Singmann et al. 2021), “emmeans” (Lenth 2022), and “ggplot2” (Wickham 2016).

#### 2.6.2 Oscillatory Data

Following the method outlined by Klimesch et al. (1998), phase‐locked activity was eliminated by subtracting the ERP average over trials from each individual trial for every participant and condition. The frequency power spectrum was then calculated for a range of 1–40 Hz on single‐trial data using convolution in the frequency domain. A Hann‐tapered window of 200 ms was applied, shifting in increments of 20 ms and 1 Hz, yielding a time resolution of ±100 ms and a spectral resolution of 5 Hz (Rayleigh frequency). The single‐trial power estimates were averaged across trials and expressed as decibel changes relative to a baseline interval from 200 to 100 ms before stimulus onset.

For explorative purposes, we computed the induced frequency power spectrum from 40 to 100 Hz in steps of 20 ms and 2 Hz. Specifically, convolution with complex Morlet wavelets with a length of 8 cycles was used, leading to a full‐width at half‐maximum (FWHM) ranging from 74 ms/8.5 Hz at 40 Hz to 30 ms/21 Hz at 100 Hz. Significant effects of interest in this frequency band are reported in Supporting Material S3.

To examine effects of personal reward, we compared the power spectra of receivers of Picture *2* in trials with a personal bonus reward (*SR_PR*) versus those with only a shared reward (*SR_noPR and SR*) using paired sample *t*‐tests at each sensor, time point and frequency bin. Furthermore, to replicate sender‐receiver differences, we compared power spectra between receivers and senders for Pictures *1 and 2*, and the selection array following Picture *1* using paired sample *t*‐tests. To correct for multiple comparisons, a cluster‐based permutation test (Maris and Oostenveld 2007) was performed for each contrast. Specifically, t‐values adjacent in sensor space, time and frequency were clustered to summed test statistics. A (sensor, time, frequency)‐sample was included in a cluster, if it had at least 3 neighboring sensors, 3 neighboring time bins (60 ms) and 2 neighboring frequency bins (2 Hz) that reached a cluster‐forming threshold of *p* < 0.05 (two‐tailed). The summed *t*‐statistics from these clusters were then compared against a Monte‐Carlo simulation of 5000 randomly bootstrapped permutations of trials. The significance probability was determined by calculating the proportion of random partitions yielding a larger test statistic than the empirically observed one. The minimum possible *p*‐value was thus *p* = 0.0002 in case of no bootstrapped test statistic being larger. Importantly, the statistical significance of this test only applies to the overall observed effect between conditions but not to the observed localization of the cluster in space, time, and frequency dimensions (Sassenhagen and Draschkow 2019). Statements about these parameters serve as descriptive information only. As in Flösch et al. (2024b), and to minimize the risk of detecting spurious effects in small, adjacent cluster‐forming data samples, a one‐sided cluster‐level alpha threshold of *p* < 0.005 was used to determine statistical significance. To illustrate the individual oscillatory effects, mean single‐subject data were averaged over the cluster dimensions and weighted by the number of significant channels, time points, and frequencies. Cohen's d for paired samples was calculated to illustrate the strength of significant cluster effects ($t/\sqrt{n}$; D. Lakens 2013).

#### 2.6.3 ERP Data

ERP mean waveforms were calculated for all experimental conditions, sensors, and participants and were baseline‐adjusted to 100 ms pre‐stimulus onset.

##### 2.6.3.1 Personal Reward Effects

Amplitudes of receivers of Picture 2 in trials yielding an actual bonus reward (*SR_PR*) were compared with trials where players did not receive a bonus (*SR_noPR and SR*) using paired sample *t*‐tests at each sensor and time point from 0 to 1000 ms post‐stimulus onset. Correction of multiple comparisons was conducted in parallel to oscillatory data (see above), that is, a cluster‐based permutation test was performed, requiring (sensor, time)‐samples to have at least three neighboring sensors and nine neighboring time bins (9 ms) that reached a cluster‐forming threshold of *p* < 0.05 (two‐tailed). The summed *t*‐statistic was compared against a Monte‐Carlo simulation of 5000 permutations, yielding a minimum possible *p*‐value of 0.0002. ERP cluster effects were visualized by weighting and averaging mean single‐subject data over significant cluster dimensions and calculating Cohen's *d* to illustrate the strength of effects ($t/\sqrt{n}$).

##### 2.6.3.2 Replicating Sender/Receiver Effects

Building upon the findings of Flösch et al. (2024a, 2024b), we focused on replicating a posterior negativity as well as P3 and late positive components. We utilized identical occipito‐temporal and centro‐parietal sensor clusters as well as similar time windows. Specifically, we scored a posterior negativity from 150 to 200 ms (cf. Flösch et al. 2024a) using the EGI sensors 95, 96, 97, 98, 104, 105, 106, 107, 108, 109, 112, 113, 114, 115, 116, 117, 118, 120, 121, 122, 123, 124, 125, 126, 127, 133, 134, 135, 136, 137, 138, 139, 140, 145, 146, 147, 148, 149, 150, 151, 152, 156, 157, 158, 159, 160, 161, 165, 166, 167, 168, 169, 170, 174, 175, 176, 177, 178, 187, 188, and 189 (see Figure 6a). Furthermore, we scored a P3 peak from 300 to 400 ms and a subsequent late positive wave from 400 to 600 ms (cf. Flösch et al. 2024a, 2024b) using the EGI sensors 6, 7, 8, 9, 16, 17, 24, 43, 44, 45, 52, 53, 60, 66, 78, 79, 80, 81, 87, 88, 89, 90, 99, 100, 101, 109, 110, 117, 118, 119, 126, 127, 128, 129, 130, 131, 132, 139, 140, 141, 142, 143, 144, 153, 154, 155, 164, 184, 185, 186, 197, 198, 207, and 257 (see Figure 6b).

Mean ERP data from respective sensor clusters and time intervals were entered in repeated measures ANOVAs including the factors *Communication* (Receiver vs. Sender), *Picture* (Picture 1 vs. Picture 2), *Trial Type* (SR vs. SR_noPR), and for P3‐related effects an additional factor *Time* (300–400 vs. 400–600 ms). Effect sizes were estimated by generalized and partial Eta‐squared. Post hoc contrasts of interest were calculated two‐tailed and FDR‐corrected familywise using the Benjamini‐Yekutieli α‐level adjustment (Benjamini and Yekutieli 2001). Effect sizes for contrasts were calculated using Cohen's *d* for paired sample comparisons ($t/\sqrt{n}$).

### 2.7 Data Reproducibility

Aggregated data and analysis scripts required to reproduce the findings are available at https://doi.org/10.48606/f2g5kr6u63djbk37. The repository includes preprocessed and averaged ERP and time–frequency data for all conditions, sensors, and participants, along with code to reproduce the cluster‐based permutation tests and ROI analyses. We also provide an adapted implementation of the FieldTrip cluster‐based permutation test that allows control over the minimum number of neighboring time points and frequencies required for cluster formation (see above). Code used to generate the time–frequency cluster figures is available at https://github.com/kpm‐floesch/niceTFRplot.

## 3 Results

### 3.1 Personal Reward Effects

The Pacman Game used a monetary gamble to enhance the personal relevance of the shared goal (see Figure 1). In half of the game trials, monitoring receivers (Picture 2) had a 50% chance to obtain a personal reward, which was granted when Picture 2 displayed a directional cue associated with a personal reward (25% trials with shared reward and personal reward; *SR_PR*) and withheld when Picture 2 displayed a directional cue that was *not* associated with a personal reward (25% trials with shared reward and no personal reward; *SR_noPR*). In the remaining game trials, no personal reward was attainable (50% trials with shared reward only; *SR*). These three conditions were contrasted in the time and frequency domains.

#### 3.1.1 Brain Oscillations 1–40 Hz

Non‐phase‐locked neural activity was examined using pairwise *t*‐tests with cluster‐based permutation correction for multiple comparisons.

##### 3.1.1.1 *SR_PR* vs. *SR_noPR*

**FIGURE 2:**
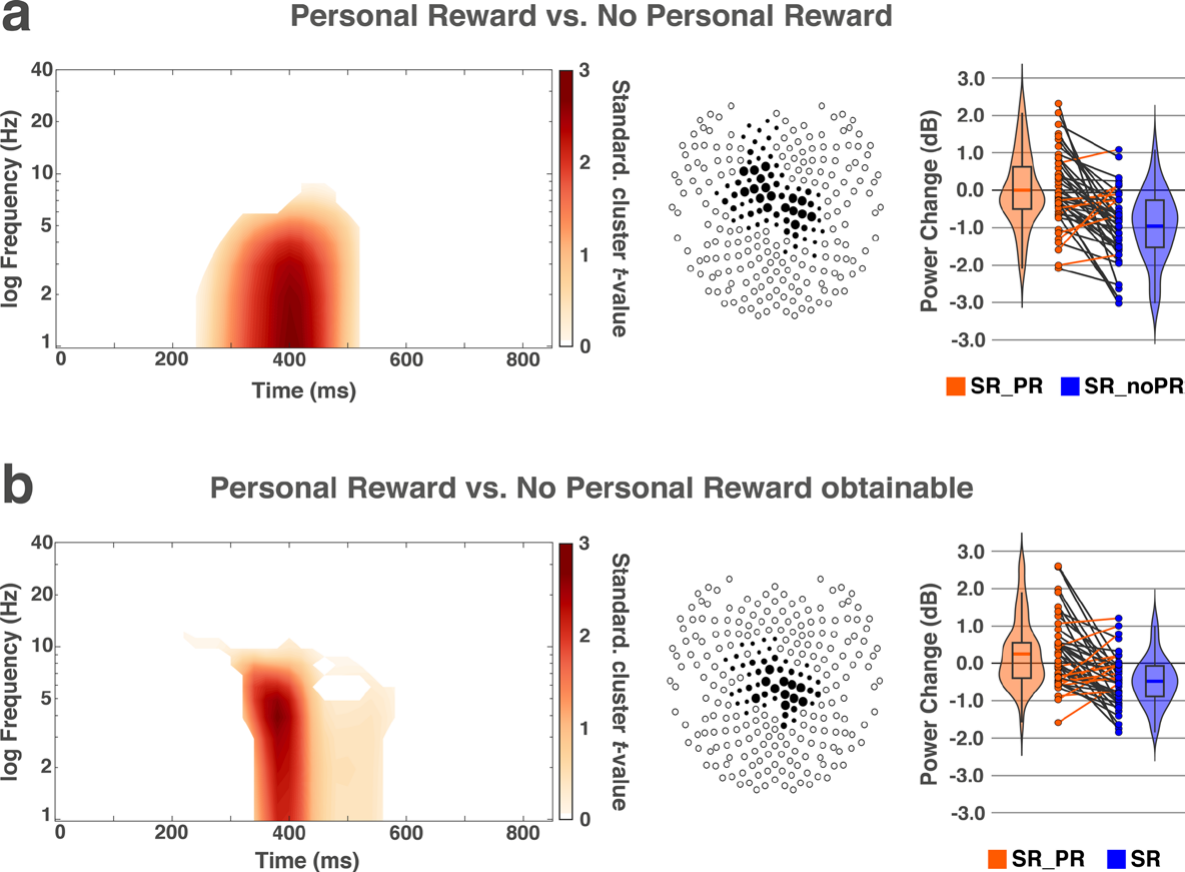
Effects of personal reward on induced oscillations (1–40 Hz), comparing trials in which monitoring receivers (Picture 2) obtained a personal reward (*SR_PR*) with (a) trials where a personal reward was attainable but not received (*SR_noPR*) and (b) trials where no additional bonus could be obtained (*SR*). Only correct trials were analyzed, ensuring that a shared reward was received in all three trial types. *Left*. Time‐frequency plots of *t*‐values illustrate the significant clusters aggregated over sensors. The summed cluster *t*‐value at each time × frequency point is standardized by dividing it by the number of significant sensors within the respective cluster. Data points not significant at cluster level are masked. Note that the statistical significance of cluster‐based permutation tests applies to the overall observed effect between conditions but not to the specific localization in space, time, and frequency. *Middle*. The topography of the cluster is shown in the display of the sensor layout (nose pointing up) with the size of the marked sensors being proportional to their contribution to the cluster. For more details about the spatial extension of the frequency clusters please refer to Figure S9. *Right*. Violin plots show individual cluster averages expressed as power change (to baseline) in decibel across time, frequency, and sensors as a function of role. Cluster averages of each subject are weighted within time, frequency, and sensor dimensions. Paired measures of all 48 participants are traced by gray lines when exhibiting the direction of effect observed by the cluster‐based statistic (see left) and orange lines when exhibiting a reversed pattern. Colored center lines within box plots represent the mean, edges mark the 25th and 75th percentiles, whiskers denote 1.5 times the interquartile range.

Contrasting *SR_PR* and *SR_noPR* trials isolates neural oscillatory effects associated with receiving a personal reward. As shown in Figure 2a, receivers obtaining a personal reward exhibited significantly greater delta to theta power (~1–7 Hz) over central and frontal sensor sites from approximately 250–500 ms (*p* = 0.0014; *d* = 0.72; see also Figure S9) in comparison to receivers who did not receive one.

Follow up analyses revealed that the delta/theta power did not differ from baseline in the *SR_PR* condition, *t*(47) < 0.01, *p* = 0.99 ns, Δ < 0.01 dB. In contrast, receivers in the SR_noPR condition showed a significant decrease in delta and theta power, *t*(47) = −5.75, *p* < 0.0001, *d* = 0.83; Δ = −1.09 dB.

No negative cluster reached significance (*p*s ≥ 0.89).

##### 3.1.1.2 *SR_PR* vs. *SR*

Comparing the *SR_PR* condition with trials in which no personal reward was possible (*SR*) serves as an internal replication that allows to evaluate the robustness of personal reward effects. Corroborating the *SR_PR* vs. *SR_noPR* contrast, we observed a positive cluster spanning from ~1 to 10 Hz over central regions from about 300 to 550 ms (*p* = 0.008; *d* = 0.62; see Figure 2b and Figure S10), which approached the prespecified significance level of *p* < 0.005 used for cluster permutation tests.

Follow up analyses indicated that receivers in the *SR* condition exhibited a decrease in delta/theta power relative to baseline, *t*(47) = −3.89, *p* = 0.0003, *d* = 0.56; Δ = −0.60 dB, whereas receivers in the *SR_PR* condition showed a slight increase, *t*(47) = 1.95, *p* = 0.056, *d* = 0.28; Δ = 0.26 dB.

No negative cluster reached significance (*p*s ≥ 0.21).

##### 3.1.1.3 *SR_noPR* vs. *SR*

Contrasting trials in which a personal reward was possible but not obtained (*SR_noPR*) with trials in which no personal reward was possible (*SR*) allowed assessment of effects related to expectancy. Neither a positive (*p* = 0.78) nor a negative cluster (*p* = 0.50) reached significance.

#### 3.1.2 Event‐Related Potentials

Phase‐locked neural activity was examined using pairwise *t*‐tests with cluster‐based permutation correction for multiple comparisons. An additional region‐ and time‐of‐interest analysis separately quantifying the RewP, P3, and LPP components is provided in Figure S4.

##### 3.1.2.1 *SR_PR* vs. *SR_noPR*

**FIGURE 3:**
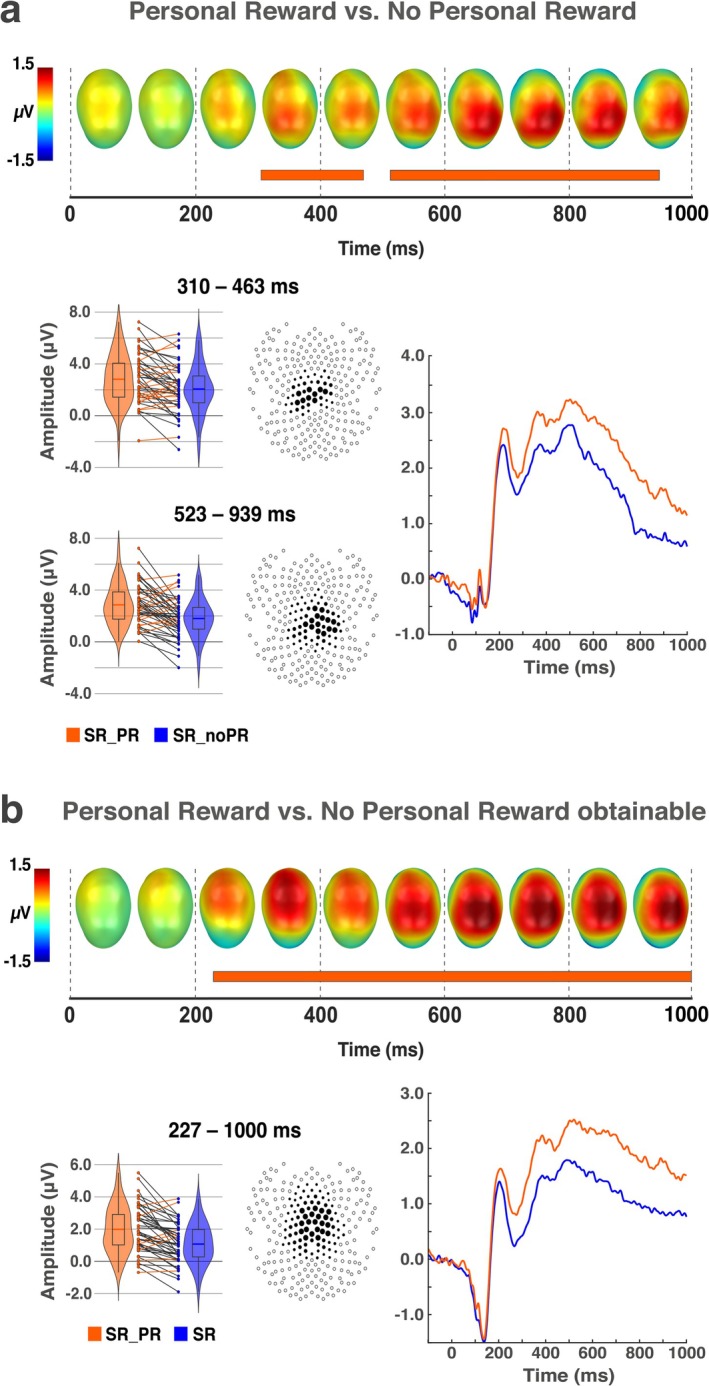
Effects of personal reward on ERPs, comparing trials in which monitoring receivers (*Picture 2*) obtained a personal reward (*SR_PR*) with (a) trials where a personal reward was attainable but not received (*SR_noPR*) and (b) trials where no additional bonus could be obtained (*SR*). Only correct trials were analyzed, ensuring that a shared reward was received in all three trial types. (*Top panels*) Scalp difference maps illustrate the personal reward effect in monitoring receivers. Colored bars over the timeline represent time windows where significant clusters occurred. Note that the statistical significance of cluster‐based permutation tests applies to the overall observed effect between conditions but not to the specific localization in space and time. (*Bottom panels*) Violin plots show individual averages across time and sensors as a function of receiver role for the cluster comparisons of *SR_PR* with *SR_noPR* (a) and *SR* (b) trials. Cluster averages of each subject are weighted within time and sensor dimensions. The topography of the cluster is shown in the display of the sensor layout (nose pointing up) with the size of the marked sensors being proportional to their contribution to the cluster. Grand‐mean ERP waveforms from the respective clusters are averaged over participants, and zero‐phase lowpass‐filtered at 14 Hz. Please note that ERP waveforms in (a) are averaged over both clusters for illustrative purposes.

Contrasting *SR_PR* and *SR_noPR* trials isolates ERP effects associated with receiving a personal reward. As shown in Figure 3a, scalp difference maps reveal that receivers obtaining a personal reward exhibited greater P3 amplitudes followed by a late positive potential over centro‐parietal regions. Supporting this notion, two significant positive clusters were observed (*p*s = 0.0310 and 0.0022, *d*s = 0.56 and 0.70) from 310 to 463 ms and from 523 to 939 ms post stimulus onset, respectively.

No negative cluster reached significance (*p*s > 0.20).

##### 3.1.2.2 *SR_PR* vs. *SR*

Comparing the *SR_PR* condition with trials in which no personal reward was possible (*SR*) serves as an internal replication that allows to evaluate the robustness of personal reward effects. Corroborating the *SR_PR* vs. *SR_noPR* contrast, we found a significant positive cluster (*p* = 0.0002, *d* = 0.90) extending over centro‐parietal to frontal sensor sites and from 227 to 1000 ms post stimulus onset. Topography and time course of the cluster are very similar: We observed again enhanced P3 amplitudes as well as a late positive potential for receivers in the SR_PR condition. In addition, this cluster also revealed a pronounced fronto‐central RewP effect from ~200–400 ms.

No negative cluster reached significance (*p*s > 0.051).

##### 3.1.2.3 *SR_noPR* vs. *SR*

Contrasting trials in which a personal reward was possible but not obtained (*SR_noPR*) with trials in which no personal reward was possible (*SR*) allowed assessment of effects related to expectancy. Neither a positive (*p* = 0.17) nor a negative cluster (*p* = 0.61) reached significance.

### 3.2 Sender‐Receiver Differences

To replicate our previous findings regarding sender‐receiver asymmetries (Flösch et al. 2024a, 2024b), we excluded trials in which a personal reward was obtained. Furthermore, as no significant differences were observed in sender‐receiver effects between the *SR_noPR* and *SR* conditions, we combined these trials for the analyses reported here (see Supporting Material S1 and S4 for a full report of findings). Notably, for both Pictures 1 and 2, we replicated previous sender‐receiver differences in the domain of induced brain oscillations (alpha to lower beta power) and event‐related potential components (early negativity, P3, and late positivity).

#### 3.2.1 Brain Oscillations 1–40 Hz

##### 3.2.1.1 Picture 1

**FIGURE 4:**
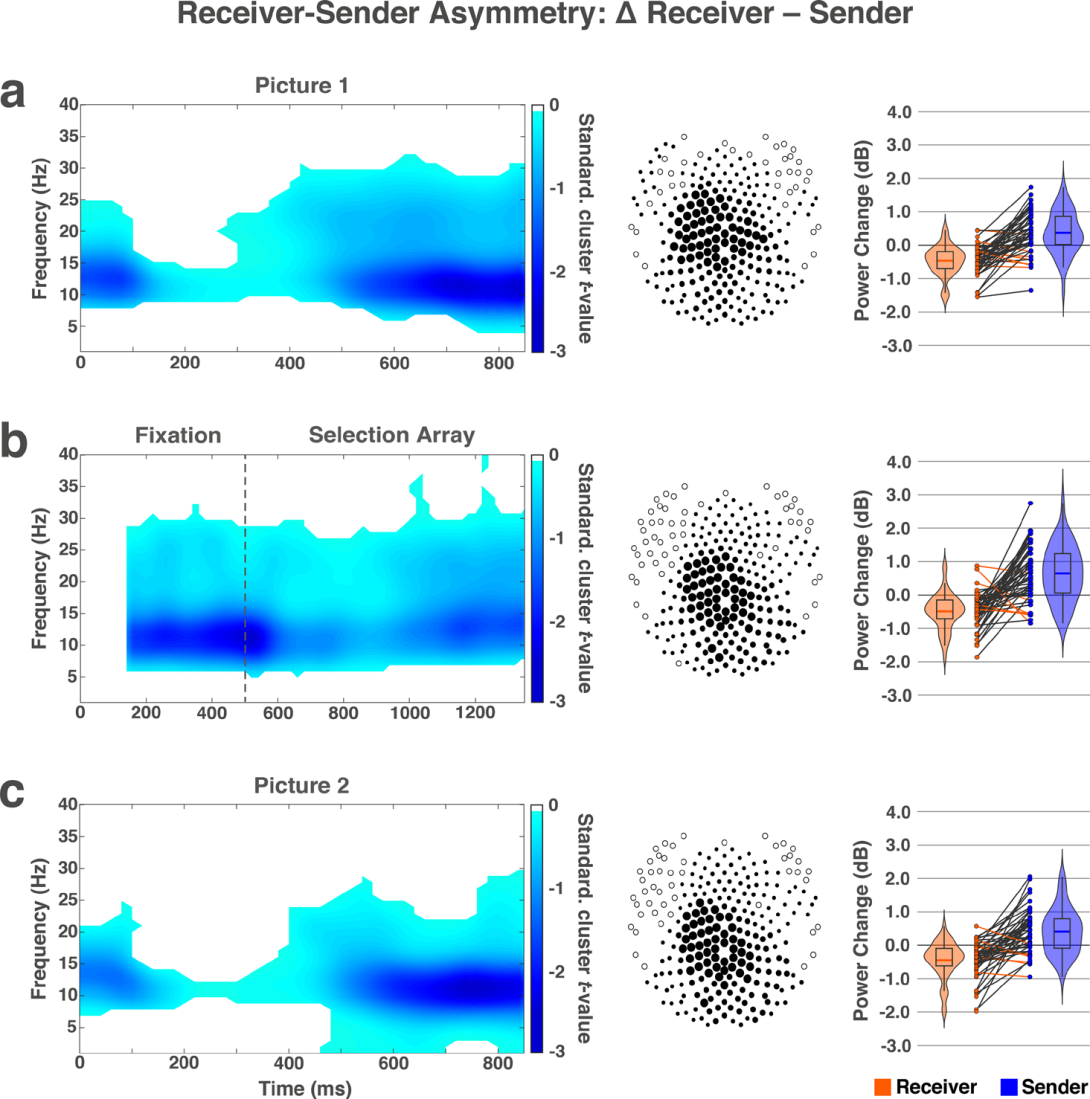
Illustration of sender‐receiver roles differences in induced oscillations (1–40 Hz) for selecting receivers (Picture 1) and monitoring receivers (Picture 2), respectively. Sender‐receiver role differences in induced oscillations were observed (a) during the presentation of the first picture cue (1000 ms) and (b) for a prolonged time spanning the fixation cross (500 ms) and the first second of the second picture selection array (500–1400 ms), using a baseline interval from 200 to 100 ms before onset of Picture 1. (c) A similar effect was found during the presentation of the second picture cue (1000 ms). For separate illustrations of *SR* and *SR_noPR* trials please refer to Supporting Material S1. For more details about the spatial extension of the frequency clusters please refer to Figures S5–S8.

As shown in Figure 4a, receiving a task‐relevant picture cue elicited a significant decrease of alpha/beta power in selecting receivers compared to senders (*p* = 0.0002; *d* = −1.14) in a widespread cluster, most pronounced over central and parietal areas, and in the alpha band from ~8 to 15 Hz. The cluster effect already begins at stimulus onset, subsides for about 300 ms, and reappears at ~400 to 850 ms.^2^

##### 3.2.1.2 Fixation and Selection Array

Figure 4b indicates that receiver‐sender differences persisted beyond the offset of picture presentation during the fixation period (0–500 ms) as well as during the first second of the selection array (500–1400 ms) with similar characteristics in topography and frequency space (*p* = 0.0002; *d* = −1.02).

##### 3.2.1.3 Picture 2

The second picture cue was informative about the partner's decision and elicited a significant ERD effect in monitoring receivers compared with senders (*p* = 0.0002; *d* = −0.98; see Figure 4c) most pronounced over central and parietal regions and within the alpha/beta band (~8–15 Hz). The temporal characteristics were similar to the alpha/beta ERD observed for Picture 1.

#### 3.2.2 Event‐Related Potentials

##### 3.2.2.1 Posterior Negativity (140–200 ms)

**FIGURE 5:**
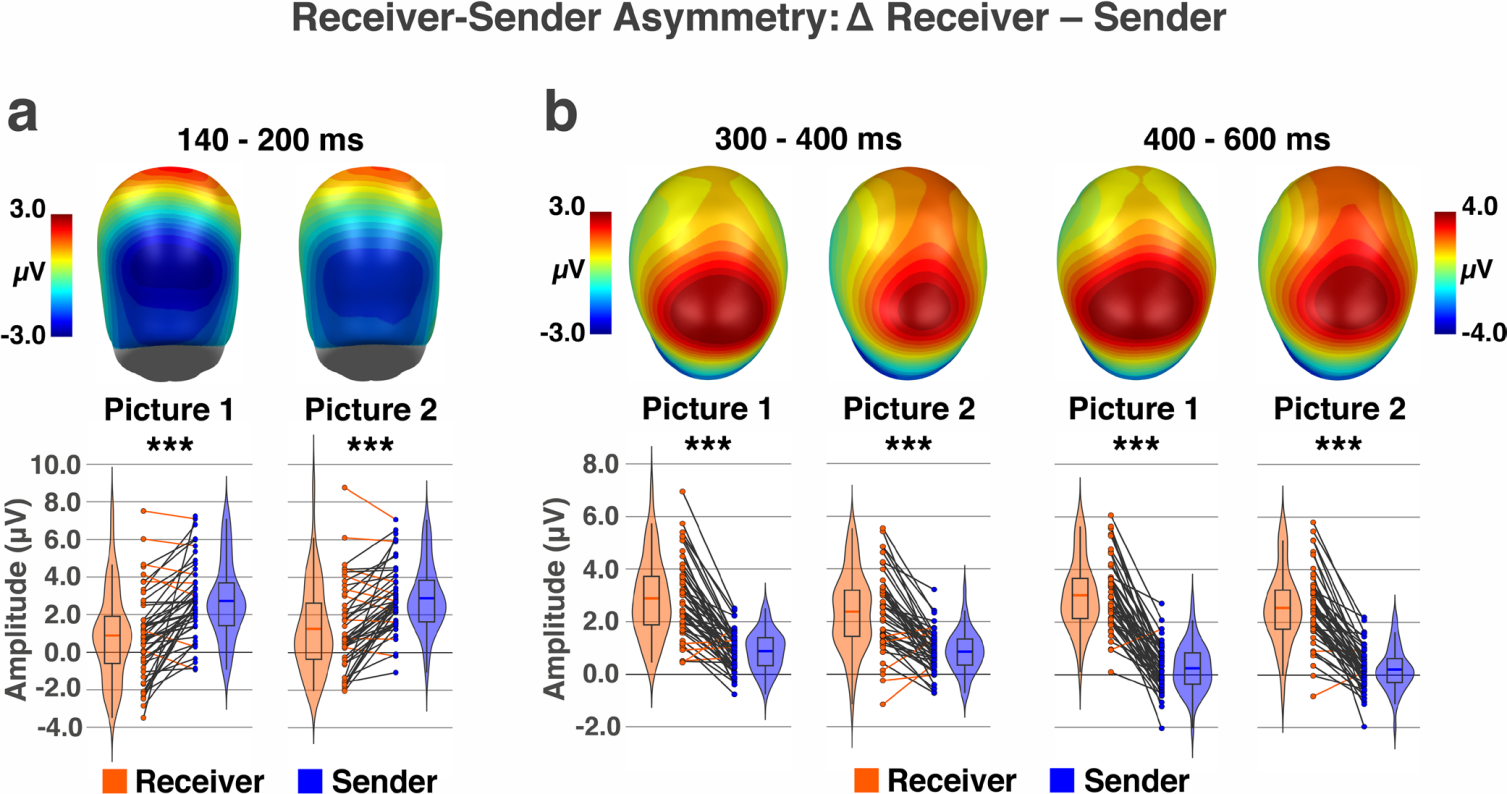
Illustration of sender‐receiver role differences associated with the posterior negativity, the P3 component (300–400 ms) and the late positivity (400–600 ms). Scalp difference maps (receiver—sender; (a) back view; (b) top view) indicate that larger amplitude deflections were found for selecting receivers (Picture 1) and monitoring receivers (Picture 2) as compared to respective senders of picture cues (a) from 140 to 200 ms at occipito‐temporal sensor sites and (b) from 300 to 600 ms over centro‐parietal areas.

**FIGURE 6:**
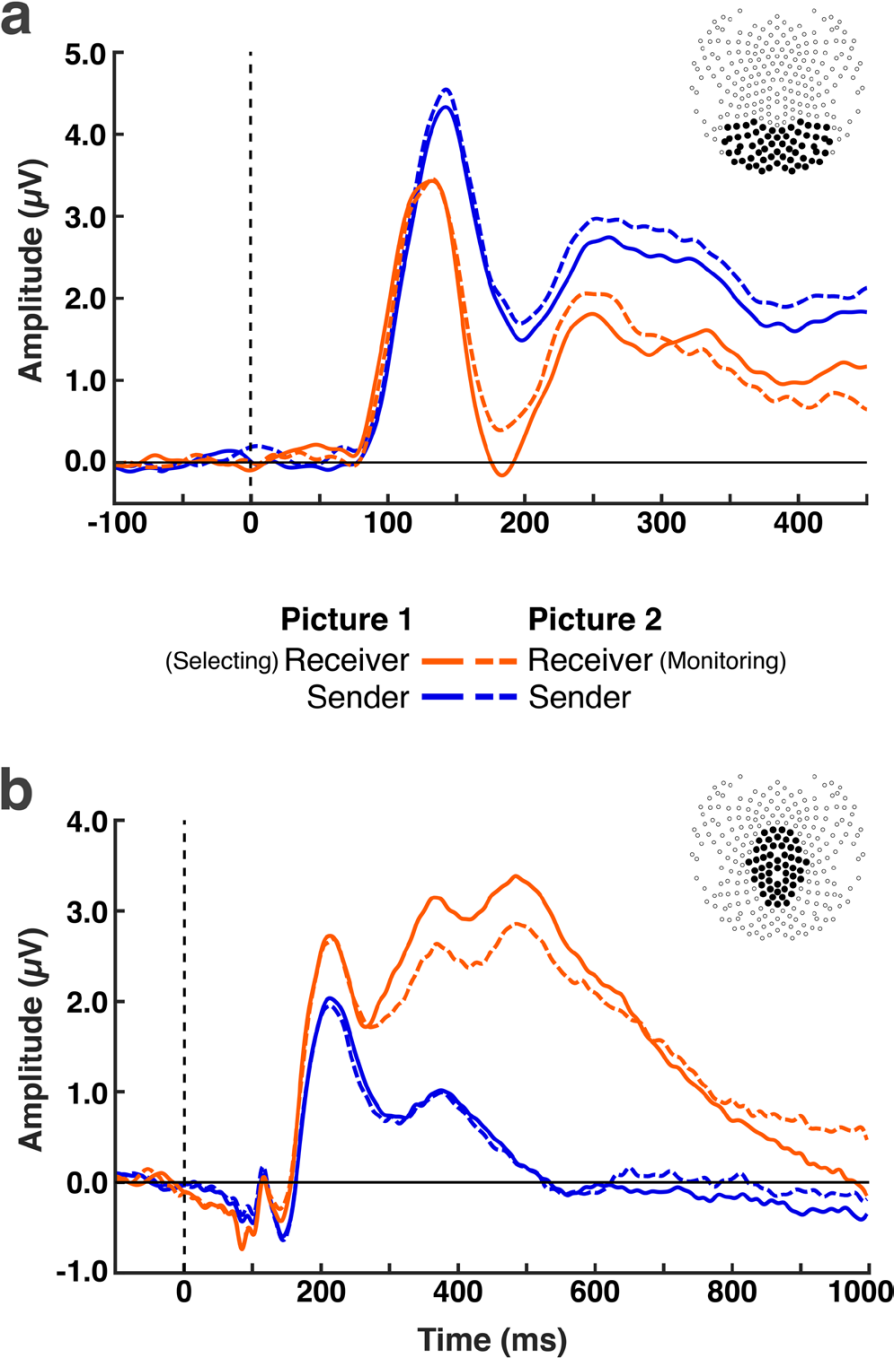
Illustration of sender‐receiver roles differences in ERPs for selecting receivers (Picture 1) and monitoring receivers (Picture 2) in an occipito‐temporal (a) and a centro‐parietal (b) sensor cluster, respectively. (a) receivers compared with senders show a relative negative deflection at occipito‐temporal sensor sites. (b) receivers compared with senders show an enhanced P3 component (300–400 ms) and subsequent late positivity (400–600 ms) at centro‐parietal sensor sites. For separate illustration of *SR* and *SR_noPR* trials please refer to Figure S3.

A two‐way ANOVA revealed a significant main effect of *Communication*, *F*(1,47) = 45.70, *p* < 0.0001, $\eta_{g}^{2}$ = 0.14, $\eta_{p}^{2}$ = 0.49, Δ = −1.73 μV, 95% CI = [−2.25, −1.21], indicating a more pronounced negativity for receivers (*M* = 1.08 μV, SE = 0.33) as compared to senders (*M* = 2.81 μV, SE = 0.27; see Figures 5a and 6a). Furthermore, a significant main effect of Picture, *F*(1,47) = 11.15, *p* = 0.002, $\eta_{g}^{2}$ = 0.004, $\eta_{p}^{2}$ = 0.19, Δ = −0.27 μV, 95% CI = [−0.43, −0.11], showed a greater negativity for Picture 1 (*M* = 1.81 μV, SE = 0.28) than Picture 2 (*M* = 2.08 μV, SE = 0.27). The two‐way interaction of Communication by Picture was not significant, *F*(1,47) = 1.15, *p* = 0.29 ns. Exploratory post hoc testing (FDR corrected for 6 tests; see Figure 5a), however, suggested that the difference between picture cues (Picture 1 vs. Picture 2) was driven only by receivers, *t*(47) = −2.85, *p* = 0.019, *d* = −0.41; Δ = −0.38 μV, 95% CI = [−0.74, −0.01], and not by senders, *t*(47) = −1.23, *p* = 0.55 ns, Δ = −0.16 μV.

##### 3.2.2.2 P3 (300–400 ms)

A significant main effect of *Communication*, *F*(1,47) = 98.12, *p* < 0.0001, $\eta_{g}^{2}$ = 0.36, $\eta_{p}^{2}$ = 0.68, Δ = 1.76 μV, 95% CI = [1.40, 2.12], indicated larger P3 amplitudes for receivers (*M* = 2.63 μV, SE = 0.21) compared with senders (*M* = 0.87 μV, SE = 0.11; see Figures 5b and 6b). The main effect of *Picture* was significant, *F*(1,47) = 13.26, *p* = 0.0007, $\eta_{g}^{2}$ = 0.01, $\eta_{p}^{2}$ = 0.22, Δ = 0.27 μV, 95% CI = [0.12, 0.41], with a greater positivity for Picture 1 (*M* = 1.88 μV, SE = 0.14) than Picture 2 (*M* = 1.62 μV, SE = 0.15). Both main effects were qualified by a two‐way interaction, *F*(1,47) = 10.70, *p* = 0.002, $\eta_{g}^{2}$ = 0.01, $\eta_{p}^{2}$ = 0.19.

Follow‐up post hoc testing (FDR corrected for six tests; see Figure 5b) revealed significant sender‐receiver differences for Picture 1, *t*(47) = 10.18, *p* < 0.0001, *d* = 1.47; Δ = 2.02 μV, 95% CI = [1.47, 2.56], and Picture 2, *t*(47) = 8.06, *p* < 0.0001, *d* = 1.16; Δ = 1.48 μV, 95% CI = [0.98, 1.99]. Confirming the hypothesis of receiver role effects, we found an increased P3 component for selecting as compared to monitoring receivers, *t*(47) = 4.17, *p* = 0.0004, *d* = 0.60; Δ = 0.50 μV, 95% CI = [0.17, 0.84]. As expected, there was no significant effect for senders, *t*(47) = 0.36, *p* = 0.99 ns, Δ = 0.03 μV.

##### 3.2.2.3 Late Positivity (400–600 ms)

Significant main effects of *Communication* (*F*(1,47) = 191.02, *p* < 0.0001, $\eta_{g}^{2}$ = 0.56, $\eta_{p}^{2}$ = 0.80, Δ = 2.56 μV, 95% CI = [2.18, 2.93]; receivers*: M* = 2.79 μV, SE = 0.19; senders: *M* = 0.23 μV, SE = 0.12) as well as of *Picture* (*F*(1,47) = 8.92, *p* = 0.004, $\eta_{g}^{2}$ = 0.01, $\eta_{p}^{2}$ = 0.16, Δ = 0.25 μV, 95% CI = [0.08, 0.42]; Picture 1: *M* = 1.63 μV, SE = 0.13; Picture 2: *M* = 1.38 μV, SE = 0.13) were qualified by a significant two‐way interaction, *F*(1,47) = 8.68, *p* = 0.005, $\eta_{g}^{2}$ = 0.01, $\eta_{p}^{2}$ = 0.16 (see Figures 5b and 6b).

Post hoc testing (FDR corrected for six tests; see Figure 5b) showed significant sender‐receiver differences for Picture 1, *t*(47) = 13.84, *p* < 0.0001, *d* = 2.00; Δ = 2.74 μV, 95% CI = [2.20, 3.29], and Picture 2, *t*(47) = 11.91, *p* < 0.0001, *d* = 1.72; Δ = 2.32 μV, 95% CI = [1.79, 2.86]. Moreover, we found again a larger late positivity for selecting compared with monitoring receivers, *t*(47) = 3.43, *p* = 0.0037, *d* = 0.50; Δ = 0.46 μV, 95% CI = [0.09, 0.83], but no effect for senders, *t*(47) = 0.52, *p* = 0.99 ns, Δ = 0.04 μV.

## 4 Discussion

Cooperation is a fundamental aspect of human interaction, enabling individuals to pursue shared goals through complementary roles and coordinated actions (Henrich 2016; Searle 1995). Unfolding over time, cooperative interactions give rise to differentiated role‐taking and strategic problem solving (Knoblich et al. 2011; Vesper et al. 2011). Experimental games offer a controlled yet socially meaningful framework to investigate these processes while preserving key elements of real‐world interaction (Schilbach et al. 2013). Using the Pacman Game, we replicated established sender–receiver differences in informational value, reflected in modulations of alpha to beta oscillations as well as early negative and late positive ERP components. Extending this paradigm, we introduced a personal reward to examine how shared and individual incentives interact during cooperation. When task‐related cues signaled an additional personal reward, they elicited key brain signatures of reward processing, including enhanced delta to theta band activity and a sustained positive potential both over centro‐parietal to frontal regions. Together, these findings demonstrate that oscillations and event‐related potentials can effectively track both role‐dependent cognitive task demands and motivational reward processing as they unfold during cooperative interaction.

In everyday life, cooperation is often intertwined with personal goals and motivations. Consequently, when pursuing a shared goal, the same outcome may hold greater motivational significance for one collaborator than for another. For instance, consider two friends planning a charity event: one is deeply invested because the cause aligns with their personal values, while the other treats it more as a casual opportunity for socializing. In this case, the success of the event holds far greater personal meaning for the first friend, as achieving the shared goal is closely tied to a personal reward. The present study incorporated this asymmetry by designing a dyadic game centered on a shared task, while also introducing elements of a personal gamble (i.e., random rewards) to examine how personal and shared incentives interact. Specifically, for each player, an idiosyncratic subset of directional cues led to a personal reward in addition to the shared reward. The game was structured so that the personal reward occurred only in correct trials and was therefore presented exclusively as the second picture. Processing of the personal reward cue was associated with a significant delta to theta power increase from approximately 300–500 ms over frontal and central sensor sites, compared to trials with only a shared reward. This finding aligns with previous dual‐choice gambling studies reporting enhanced delta and theta activity approximately 200–600 ms after feedback onset, associated with reward magnitude and expectancy. (Cohen et al. 2007, 2009; Marco‐Pallarés et al. 2008; Williams et al. 2021). However, rather than informing participants about the reward through an explicit feedback cue (e.g., a smiley), participants had to infer the personal reward based on the identity of Picture 2. This resembles everyday situations, where explicit feedback is infrequent, and individuals rely on contextual cues to infer personal outcomes.

Personal reward processing also modulated event‐related potentials. When monitoring receivers obtained a personal reward (*SR_PR*) compared to trials where they had a chance to win but did not receive it (*SR_noPR*), they exhibited a pronounced positivity over centro‐parietal regions beginning around 230 ms and persisting throughout the picture presentation (1000 ms). This pattern of finding aligns with prior research showing larger P3 and LPP components when participants received positive feedback in non‐social gambling tasks (Glazer et al. 2018; Walsh and Anderson 2012). Furthermore, a similar effect was found when comparing personal reward trials (*SR_PR*) with trials yielding no chance to win a personal reward (*SR*). This cluster showed a topographical shift from anterior to centro‐parietal regions, consistent with a fronto‐central RewP from ~230 to 400 ms (see Proudfit 2015). Overall, findings from both non‐phase‐locked brain oscillations and phase‐locked ERPs demonstrate sensitivity to a personal reward beyond the reception of a shared reward.

Reinforcement learning theory emphasizes the role of prediction errors—discrepancies between expected and actual outcomes—in guiding behavior and optimizing decision‐making (Lee et al. 2012; Proudfit 2015; Sambrook and Goslin 2015; Walsh and Anderson 2012). Prediction errors encompass both outcome valence and surprise (i.e., unexpectedness). The present design allows us to disentangle these components. Participants could infer whether a personal reward was possible based on the direction the Pacman had to move, thereby forming expectations about the upcoming outcome. By comparing *SR_PR* and *SR_noPR* trials, we isolate the effect of outcome valence, as both conditions involve the same level of surprise. Conversely, comparing the *SR_noPR* and *SR* conditions isolates the effect of surprise, as these conditions differ in expectation but result in the same (personally non‐rewarding) outcome. Our results indicate that reward‐related neural activity in our paradigm reflects outcome valence but not surprise, as the latter contrast showed no significant differences in either the time or frequency domain. Exploratory Bayes analyses indicated anecdotal to moderate evidence for the null hypothesis. This finding aligns with previous research suggesting that surprise processing is attenuated or even suppressed when personal reward is not contingent on player performance and thus cannot be leveraged for learning and adaptation (Wurm et al. 2021).

Future research could extend these findings by comparing shared and personal rewards that are directly dependent on task performance. For example, obtaining a personal reward in the role of the monitoring receiver could be made contingent on the participant's initial choice as sender of the first picture cue. In this scenario, players could maximize personal reward by learning the probabilistic relationship between the initial choice and subsequent outcome. Surprise would no longer be tied to the experimental conditions but would instead vary across trials as a function of experience‐driven learning, as predicted by reinforcement‐learning models. Accordingly, posterior parietal activity should track this surprise signal and relate to the adaptation and optimization of choice behavior (Nassar et al. 2019; Wurm et al. 2021). Moreover, in the present design, the opportunity to obtain a personal reward was linked to the second picture and was available only to the receiver. This design allowed us to dissociate motivational relevance (Picture 2) from task‐related processing demands (Picture 1). Building on this approach, future research could implement choice‐contingent reward structures and systematically vary the association between reward availability and player role to further disentangle role‐related and reward‐related effects.

Our design allows us to differentiate the influence of valence and surprise on feedback‐related brain activity. At the same time, the more naturalistic character of our task entails more complex rules, greater task demands, and more complex feedback stimuli than classical feedback paradigms. Importantly, these variables do not differ between the conditions of interest, particularly not between the *SR_PR* and *SR_noPR* conditions, where feedback‐related differences were observed. Nevertheless, they may have contributed to a pattern of feedback‐related activity that diverges from that typically reported in simplified tasks. For instance, feedback processing is sensitive to working memory demands (Collins and Frank 2012), and the posterior P3 in particular has been linked to context updating in working memory (Donchin and Coles 1988; San Martín 2012). Greater rule complexity and the need to maintain multiple cues in working memory may therefore have modulated feedback‐related responses, potentially resulting in attenuated or enhanced activity (Collins et al. 2017). In addition, feedback‐related brain activity elicited by our picture cues may be characterized by reduced amplitudes or increased latency variability due to greater working memory demands (Krigolson et al. 2015, 2012), richer informational content (Cockburn and Holroyd 2018), variability in feedback complexity (Baker and Holroyd 2011), reduced perceptual salience (Pfabigan et al. 2015), or higher perceptual variability (Ernst and Steinhauser 2020). Thus, while the present study demonstrates that feedback‐related brain activity generalizes from simple and highly controlled tasks to a more naturalistic context, future research could examine how these factors influenced the present results.

Experimental games offer the advantage that their rules can be adapted to investigate additional processes while maintaining core features that ensure replicability of findings. In this study, we introduced personal reward while preserving key aspects of the Pacman Game related to sender‐receiver effects, thereby allowing for a conceptual replication of our previous findings (cf. Lakens 2022; Sidman 1960). One set of replication findings concerns brain oscillations linked to sender‐receiver differences. Based on Picture 1, selecting receivers had to semantically process the symbolic directional cue, retrieve the corresponding cues from memory, maintain them in working memory, and select the appropriate cue from the search array. Replicating earlier work (Flösch et al. 2024b), selecting receivers showed a broad alpha/beta ERD (~7–35 Hz) mainly over centro‐parietal to frontal regions in response to Picture 1, compared to senders. This induced power decrease persisted beyond stimulus presentation into the fixation phase and the first second of the visual search array, indicating sustained stimulus processing linked to task demands. This interpretation aligns with the observation that the ERD effect is most pronounced in the upper alpha band (~10–14 Hz), a specific frequency range previously associated with semantic processing demands (Hanslmayr et al. 2009; Klimesch et al. 1997, 2005). A similar effect was observed for Picture 2. Although monitoring receivers did not perform an overt task, the cue carried high motivational relevance, as it determined the partner's decision outcome and the potential for shared and personal reward. Thus, receivers needed to represent more information than senders. Consistent with the ‘information‐via‐desynchronization’ hypothesis, pronounced alpha to beta power decreases in response to both picture cues likely reflect higher demands on information representation (Hanslmayr et al. 2012; Michelmann et al. 2022).

The present work also replicated ERP findings (Flösch et al. 2024a, 2024b). As predicted, receivers showed an enhanced posterior negativity from 140 to 200 ms in response to both picture cues compared to senders, presumably reflecting early attentional allocation to task‐relevant or motivationally salient stimuli (Hopfinger et al. 2004; Schupp et al. 2006). This was followed by a pronounced P3 component from 300 to 400 ms, indicating a higher informational value of cues for receivers than senders (Johnson 1988), and a subsequent late centro‐parietal positivity from 400 to 600 ms, associated with semantic stimulus processing (Leckey and Federmeier 2020) and memory retrieval (Münte et al. 2000). In previous research, participants showed a late positivity (P600) when retrieving semantic world knowledge (Sassenhagen and Bornkessel‐Schlesewsky 2015) or semantically encoded words (Münte et al. 2000; Paller and Kutas 1992). In the present study, Picture 1 was highly task‐relevant for selecting receivers, as they had to semantically process it and retrieve the corresponding symbol to complete the task. Accordingly, P3 and late positive amplitudes were significantly larger for Picture 1 than Picture 2, reflecting its greater task relevance. In earlier versions of the game with only two symbolic cues per direction, monitoring receivers exhibited a robust P3 but no distinct late component in response to Picture 2, suggesting that memory retrieval had already been completed before Picture 1 was sent (Flösch et al. 2024a). In contrast, the current game version employed three cues per direction, leaving two possible outcomes for Picture 2 and thereby presumably increasing semantic processing demands. Additionally, the possibility of obtaining a personal reward may have further motivated receivers to process Picture 2 more thoroughly. Future adaptations of the game could examine how task difficulty modulates these neural processes, for example, by varying the size and complexity of the cue set and thereby increasing memory load.

## 5 Conclusion

Humans are built for cooperation. At the core of the human ‘interaction engine’ (Levinson 2006) lies individual role taking. When pursuing a shared goal, people implicitly agree that each collaborator takes a role with specific tasks necessary to achieve that goal (Searle 1995; Tomasello 2019). The Pacman Game provides a naturalistic framework to trace task demands associated with such individual collaborative roles. In this respect, the present study conceptually replicated modulations in alpha to lower beta power as well as early negative and late positive ERP components as a function of affective and cognitive states related to individual player roles. Beyond replication, the present study expanded the Pacman Game to simulate cooperative situations where outcomes hold greater personal significance for one collaborator. In daily life, direct feedback is rare, and individuals often infer personal rewards or losses from social cues. Similarly, in this experiment, participants inferred their bonus reward from the second picture cue. In response to this naturalistic feedback, monitoring receivers showed increased delta to theta activity and sustained positive ERP modulations, reflecting differences in reward magnitude tied to personal rewards. In conclusion, neural measures obtained in the context of experimental games can shed light on the question of how people cooperate in naturalistic contexts. Systematically modifying the game rules allows the study of more and more complex situations, thus bridging the gap between established research paradigms and real‐world social interactions.

## Author Contributions

**Karl‐Philipp Flösch:** conceptualization, formal analysis, methodology, investigation, visualization, writing – original draft, writing – review and editing. **Harald T. Schupp:** conceptualization, funding acquisition, supervision, writing – original draft, writing – review and editing. **Marco Steinhauser:** writing – original draft, writing – review and editing. **Tobias Flaisch:** methodology, writing – review and editing.

## Funding

This research was supported by the Centre for the Advanced Study of Collective Behavior, funded by the Deutsche Forschungsgemeinschaft (DFG, German Research Foundation) under Germany's Excellence Strategy (EXC2117‐422037984 granted to H.S.).

## Conflicts of Interest

The authors declare no conflicts of interest.

## Supporting information


**Figure S1:** Illustration of sender‐receiver role differences in induced oscillations (1–40 Hz) during *SR* game trials for selecting *Receivers* (Picture 1) and monitoring *Receivers* (Picture 2), respectively. Sender‐receiver role differences in induced oscillations were traced for selecting *Receivers* (a) during presentation of the first picture cue (1000 ms) and (b) for a prolonged time spanning the fixation cross (500 ms) and the first second of the picture selection array (500–1400 ms), using the same baseline interval from 200 to 100 ms before onset of Picture 1. (c) A similar effect was found during the presentation of the second picture cue (1000 ms). *Left*. Time‐frequency plots of *t*‐values illustrate the significant clusters aggregated over sensors. The summed cluster *t*‐value at each time × frequency point is standardized by dividing it by the number of significant sensors within the respective cluster. Data points not significant at cluster level are masked. *Middle*. The topography of the cluster is shown in the display of the sensor layout (nose pointing up) with the size of the marked sensors being proportional to their contribution to the cluster. For more details about the spatial extension of the frequency clusters please refer to Figures S5–S7. *Right*. Violin plots show individual cluster averages expressed as power change (to baseline) in decibel across time, frequency, and sensors as a function of role. Cluster averages of each subject are weighted within time, frequency, and sensor dimensions. Paired measures of all 48 participants are traced by gray lines when exhibiting the direction of effect observed by the cluster‐based statistic (see left) and orange lines when exhibiting a reversed pattern. Colored center lines within box plots represent the mean, edges mark the 25th and 75th percentiles, whiskers denote 1.5 times the interquartile range.
**Figure S2:** Illustration of sender‐receiver role differences in induced oscillations (1–40 Hz) during *SR_noPR/SR_PR* game trials for selecting *Receivers* (Picture 1) and monitoring *Receivers* (Picture 2), respectively. Sender‐receiver role differences in induced oscillations were traced for selecting *Receivers* (a) during presentation of the first picture cue (1000 ms) and (b) for a prolonged time spanning the fixation cross (500 ms) and the first second of the picture selection array (500–1400 ms), using the same baseline interval from 200 to 100 ms before onset of Picture 1. (c, d) A similar effect was found during the presentation of the second picture cue (1000 ms). For a description of the different panels, please refer to Figure S1. For more details about the spatial extension of the frequency clusters please refer to Figures S5–S8.
**Figure S3:** Grand mean ERP waveforms of *Receivers* and *Senders* in an occipito‐temporal (a) and a centro‐parietal (b) sensor cluster, respectively. (a) *Receivers* compared with *Senders* show a relative negative deflection at occipito‐temporal sensor sites. (b) *Receivers* compared with *Senders* show an enhanced P3 component (300–400 ms) and subsequent late positivity (400–600 ms) at centro‐parietal sensor sites. *Receivers* obtaining a personal reward (*SR_PR*) exhibit a sustained late positive potential compared to all other player roles (600–1000 ms). ERP waveforms were calculated using the cluster as displayed in the sensor layouts (nose pointing up), zero‐phase lowpass‐filtered at 14 Hz for illustrative purposes.
**Figure S4:** Illustration of personal reward effects in monitoring receivers associated with (a) the Reward Positivity (250–400 ms), (b) the P3 component (300–450 ms), and (c) the Late Positive Potential (450–1000 ms). Scalp difference maps in top view (nose pointing up) indicate larger amplitude deflections when monitoring receivers obtained a personal reward (*SR_PR*) compared to trials in which a personal reward was attainable but not received (*SR_noPR*) and trials in which no additional bonus could be obtained (*SR*). Only correct trials were analyzed, ensuring that a shared reward was received in all three trial types. Sensor layouts depict the clusters used for analysis. All *t*‐tests were conducted two‐sided, *p*‐values are uncorrected, Cohen's *d* was calculated by 𝑡𝑡/√𝑛𝑛. In the main Results section, these data were analyzed using a cluster‐based permutation approach.
**Figure S5:** Illustration of sender‐receiver role differences in induced oscillations (1–40 Hz) for *Selecting Receivers* (Picture 1) during *SR* and *SR_noPR* trials, respectively. Time‐frequency plots of t‐values illustrate respective clusters (see S1 and S2) at each sensor site from 0 to 850 ms post stimulus onset and from 1 to 40 Hz. Non‐significant data points are masked at an alpha‐level of p = 0.025 (one‐tailed).
**Figure S6:** Illustration of sender‐receiver role differences in induced oscillations (1–40 Hz) for *Selecting Receivers* during *SR* and *SR_noPR* trials, respectively. Time‐frequency plots of *t*‐values illustrate respective clusters (see Figures S1 and S2) at each sensor site and from 1 to 40 Hz for a prolonged time spanning the fixation cross (500 ms) and the first second of the picture selection array (500–1400 ms). Non‐significant data points are masked at an alpha‐level of *p* = 0.025 (one‐tailed).
**Figure S7:** Illustration of sender‐receiver role differences in induced oscillations (1–40 Hz) for *Monitoring Receivers* (Picture 2) during *SR* and *SR_noPR* trials, respectively. Time‐frequency plots of t‐values illustrate respective clusters (see Figures S1 and S2) at each sensor site from 0 to 850 ms post stimulus onset and from 1 to 40 Hz. Non‐significant data points are masked at an alpha‐level of p = 0.025 (one‐tailed).
**Figure S8:** Illustration of sender‐receiver role differences in induced oscillations (1–40 Hz) for *Monitoring Receivers* (Picture 2) wo obtained a personal reward (*SR_PR*). Time‐frequency plots of *t*‐values illustrate respective clusters (see Figures S1 and S2) at each sensor site from 0 to 850 ms post stimulus onset and from 1 to 40 Hz. Non‐significant data points are masked at an alpha‐level of *p* = 0.025 (one‐tailed).
**Figure S9:** Illustration of role effects in induced oscillations (1–40 Hz) comparing trials where monitoring *Receivers* (Picture 2) actually won a personal reward (*SR_PR*) versus (a) trials where they could obtain but did not receive a personal reward (*SR_noPR*) and (b) trials where they could not obtain an additional bonus (*SR*). Please note that only correct trials were included in the analyses, assuring that a shared reward was received in all three trial types. Time‐frequency plots of *t*‐values illustrate respective clusters (see Figure 2 of main results) at each sensor site from 0 to 850 ms post stimulus onset and from 1 to 40 Hz. Non‐significant data points are masked at an alpha‐level of *p* = 0.025 (one‐tailed). The y‐axis shows logarithmic frequency in Hz.
**Figure S10:** Illustration of a significant negative cluster (*p* = 0.012, *d* = −0.64) in induced oscillations (1–40 Hz) for *Selecting Receivers* as compared to *Monitoring Receivers*. The pictures were presented in a time window of 0 to 1000 ms. For a description of the different panels, please refer to Figures S1 and S5.
**Figure S11:** Illustration of a significant positive cluster (*p* = 0.011, *d* = 0.58) in induced oscillations (1–40 Hz) for *Selecting Receivers* as compared to *Senders* during *SR* trials. The picture was presented in a time window of 0 to 1000 ms, followed by a fixation cross for 500 ms and the first second of the picture selection array (1500–2500 ms). For a description of the different panels, please refer to Figures S1 and S5.
**Figure S12:** Illustration of a significant positive cluster (*p* = 0.0024 and 0.008; *d = 1.16*) in induced oscillations (1–40 Hz) for *Selecting Receivers* as compared to *Senders* during *SR_noPR* trials. The picture was presented in a time window of 0 to 1000 ms, followed by a fixation cross for 500 ms and the first second of the picture selection array (1500–2500 ms). For a description of the different panels, please refer to Figures S1 and S5.
**Figure S13:** Illustration of a significant positive cluster (*p* = 0.0002*; d = 0.90*) in induced oscillations (40–100 Hz) for *Selecting Receivers* as compared to *Senders* during *SR* trials. The picture was presented in a time window of 0 to 1000 ms, followed by a fixation cross for 500 ms and the first second of the picture selection array (1500–2500 ms). For a description of the different panels, please refer to Figures S1 and S5.
**Figure S14:** Illustration of a significant positive cluster (*p* = 0.0002; *d* = 1.11) in induced oscillations (40–100 Hz) for *Selecting Receivers* as compared to *Senders* during *SR_noPR* trials. The picture was presented in a time window of 0 to 1000 ms, followed by a fixation cross for 500 ms and the first second of the picture selection array (1500–2500 ms). For a description of the different panels, please refer to Figures S1 and S5.
**Table S1:** Posterior negativity (140–200 ms): ANOVA model including the factor Trial Type (*SR* vs. *SR_noPR*).
**Table S2:** Central positivity (300–600 ms): ANOVA model including the factor Trial Type (*SR* vs. *SR_noPR*).

## Data Availability

The aggregated data and analysis scripts necessary to reproduce the findings of this study are available at https://doi.org/10.48606/f2g5kr6u63djbk37. The full data are not publicly archived, as participants did not provide consent for sharing their individual raw EEG recordings.

## Endnotes

^1^
The family of P3‐like positivities is labeled differently across research domains. Consistent with our previous work, we use *late positivity* (also referred to as LPC or P600) to denote P3‐like deflections over centro‐parietal to frontal regions in the context of semantic processing and task relevance. In the context of affective or reward processing, we use *late positive potential* (LPP) for comparable deflections. In both domains, *P3* refers to a positive peak temporally preceding these later components.

^2^
While the late effect, most pronounced from ~500 to 850 ms, conceptually replicates the results of our previous study (Flösch et al. 2024a, 2024b), the early effect (~0–200 ms) is most likely driven by the sender rather than the receiver role. For a more detailed analysis, please refer to Supporting Material S2.

## References

[psyp70341-bib-0001] Baker, T. E. , and C. B. Holroyd . 2011. “Dissociated Roles of the Anterior Cingulate Cortex in Reward and Conflict Processing as Revealed by the Feedback Error‐Related Negativity and N200.” Biological Psychology 87, no. 1: 25–34. 10.1016/j.biopsycho.2011.01.010. 21295109

[psyp70341-bib-0002] Benjamini, Y. , and D. Yekutieli . 2001. “The Control of the False Discovery Rate in Multiple Testing Under Dependency.” Annals of Statistics 29, no. 4: 998. 10.1214/aos/1013699998.

[psyp70341-bib-0003] Cockburn, J. , and C. B. Holroyd . 2018. “Feedback Information and the Reward Positivity.” International Journal of Psychophysiology 132: 243–251. 10.1016/j.ijpsycho.2017.11.017. 29208491

[psyp70341-bib-0004] Cohen, M. X. , C. E. Elger , and J. Fell . 2009. “Oscillatory Activity and Phase‐Amplitude Coupling in the Human Medial Frontal Cortex During Decision Making.” Journal of Cognitive Neuroscience 21, no. 2: 390–402. 10.1162/jocn.2008.21020. 18510444

[psyp70341-bib-0005] Cohen, M. X. , C. E. Elger , and C. Ranganath . 2007. “Reward Expectation Modulates Feedback‐Related Negativity and EEG Spectra.” NeuroImage 35, no. 2: 968–978. 10.1016/j.neuroimage.2006.11.056. 17257860 PMC1868547

[psyp70341-bib-0006] Collins, A. G. E. , B. Ciullo , M. J. Frank , and D. Badre . 2017. “Working Memory Load Strengthens Reward Prediction Errors.” Journal of Neuroscience 37, no. 16: 4332–4342. 10.1523/JNEUROSCI.2700-16.2017. 28320846 PMC5413179

[psyp70341-bib-0007] Collins, A. G. E. , and M. J. Frank . 2012. “How Much of Reinforcement Learning Is Working Memory, Not Reinforcement Learning? A Behavioral, Computational, and Neurogenetic Analysis.” European Journal of Neuroscience 35, no. 7: 1024–1035. 10.1111/j.1460-9568.2011.07980.x. 22487033 PMC3390186

[psyp70341-bib-0008] Donaldson, K. R. , B. Ait Oumeziane , S. Hélie , and D. Foti . 2016. “The Temporal Dynamics of Reversal Learning: P3 Amplitude Predicts Valence‐Specific Behavioral Adjustment.” Physiology & Behavior 161: 24–32. 10.1016/j.physbeh.2016.03.034. 27059320 PMC5426362

[psyp70341-bib-0009] Donchin, E. , and M. G. H. Coles . 1988. “Is the P300 Component a Manifestation of Context Updating?” Behavioral and Brain Sciences 11, no. 3: 357–374. 10.1017/S0140525X00058027.

[psyp70341-bib-0010] Ernst, B. , and M. Steinhauser . 2020. “The Effect of Feedback Novelty on Neural Correlates of Feedback Processing.” Brain and Cognition 144: 105610. 10.1016/j.bandc.2020.105610. 32777688

[psyp70341-bib-0011] Flösch, K.‐P. , T. Flaisch , M. A. Imhof , and H. T. Schupp . 2024a. “Dyadic Cooperation With Human and Artificial Agents: Event‐Related Potentials Trace Dynamic Role Taking During an Interactive Game.” Psychophysiology 61, no. 1: e14433. 10.1111/psyp.14433. 37681492

[psyp70341-bib-0012] Flösch, K.‐P. , T. Flaisch , M. A. Imhof , and H. T. Schupp . 2024b. “Alpha/Beta Oscillations Reveal Cognitive and Affective Brain States Associated With Role Taking in a Dyadic Cooperative Game.” Cerebral Cortex 34, no. 1: 487. 10.1093/cercor/bhad487. 38100327

[psyp70341-bib-0013] Foti, D. , A. Weinberg , J. Dien , and G. Hajcak . 2011. “Event‐Related Potential Activity in the Basal Ganglia Differentiates Rewards From Nonrewards: Temporospatial Principal Components Analysis and Source Localization of the Feedback Negativity.” Human Brain Mapping 32, no. 12: 2207–2216. 10.1002/hbm.21182. 21305664 PMC6870417

[psyp70341-bib-0014] Gallotti, M. , and C. D. Frith . 2013. “Social Cognition in the We‐Mode.” Trends in Cognitive Sciences 17, no. 4: 160–165. 10.1016/j.tics.2013.02.002. 23499335

[psyp70341-bib-0015] Glazer, J. E. , N. J. Kelley , N. Pornpattananangkul , V. A. Mittal , and R. Nusslock . 2018. “Beyond the FRN: Broadening the Time‐Course of EEG and ERP Components Implicated in Reward Processing.” International Journal of Psychophysiology 132: 184–202. 10.1016/j.ijpsycho.2018.02.002. 29454641

[psyp70341-bib-0016] Goregliad Fjaellingsdal, T. , D. Schwenke , E. Ruigendijk , S. Scherbaum , and M. G. Bleichner . 2020. “Studying Brain Activity During Word‐By‐Word Interactions Using Wireless EEG.” PLoS One 15, no. 3: e0230280. 10.1371/journal.pone.0230280. 32208429 PMC7092963

[psyp70341-bib-0017] Goregliad Fjaellingsdal, T. , D. Schwenke , S. Scherbaum , et al. 2020. “Expectancy Effects in the EEG During Joint and Spontaneous Word‐By‐Word Sentence Production in German.” Scientific Reports 10, no. 1: 5460. 10.1038/s41598-020-62155-z. 32214133 PMC7096441

[psyp70341-bib-0018] HajiHosseini, A. , A. Rodríguez‐Fornells , and J. Marco‐Pallarés . 2012. “The Role of Beta‐Gamma Oscillations in Unexpected Rewards Processing.” NeuroImage 60, no. 3: 1678–1685. 10.1016/j.neuroimage.2012.01.125. 22330314

[psyp70341-bib-0019] Hanslmayr, S. , B. Spitzer , and K.‐H. T. Bäuml . 2009. “Brain Oscillations Dissociate Between Semantic and Nonsemantic Encoding of Episodic Memories.” Cerebral Cortex 19, no. 7: 1631–1640. 10.1093/cercor/bhn197. 19001457

[psyp70341-bib-0020] Hanslmayr, S. , T. Staudigl , and M.‐C. Fellner . 2012. “Oscillatory Power Decreases and Long‐Term Memory: The Information via Desynchronization Hypothesis.” Frontiers in Human Neuroscience 6: 74. 10.3389/fnhum.2012.00074. 22514527 PMC3322486

[psyp70341-bib-0021] Henrich, J. 2016. The Secret of Our Success: How Culture Is Driving Human Evolution, Domesticating Our Species, and Making Us Smarter. Princeton University Press.

[psyp70341-bib-0022] Henrich, N. , and J. Henrich . 2007. Why Humans Cooperate: A Cultural and Evolutionary Explanation. Oxford University Press.

[psyp70341-bib-0023] Hopfinger, J. B. , S. J. Luck , and S. A. Hillyard . 2004. “Selective Attention: Electrophysiological and Neuromagnetic Studies.” In The Cognitive Neurosciences, edited by M. S. Gazzaniga , 561–574. Boston Review.

[psyp70341-bib-0024] Huizinga, J. 1956. Homo Ludens: Vom Ursprung der Kultur im Spiel. Rowohlts Enzyklopädie. Vol. 435. Rowohlt.

[psyp70341-bib-0025] Jahng, J. , J. D. Kralik , D.‐U. Hwang , and J. Jeong . 2017. “Neural Dynamics of Two Players When Using Nonverbal Cues to Gauge Intentions to Cooperate During the Prisoner's Dilemma Game.” NeuroImage 157: 263–274. 10.1016/j.neuroimage.2017.06.024. 28610901

[psyp70341-bib-0026] Johnson, R. 1988. “The Amplitude of the P300 Component of the Event‐Related Potential: Review and Synthesis.” Advances in Psychophysiology 3: 69–137.

[psyp70341-bib-0027] King‐Casas, B. , D. Tomlin , C. Anen , C. F. Camerer , S. R. Quartz , and P. R. Montague . 2005. “Getting to Know You: Reputation and Trust in a Two‐Person Economic Exchange.” Science 308, no. 5718: 78–83. 10.1126/science.1108062. 15802598

[psyp70341-bib-0028] Klimesch, W. , M. Doppelmayr , T. Pachinger , and H. Russegger . 1997. “Event‐Related Desynchronization in the Alpha Band and the Processing of Semantic Information.” Cognitive Brain Research 6, no. 2: 83–94. 10.1016/S0926-6410(97)00018-9. 9450602

[psyp70341-bib-0029] Klimesch, W. , H. Russegger , M. Doppelmayr , and T. Pachinger . 1998. “A Method for the Calculation of Induced Band Power: Implications for the Significance of Brain Oscillations.” Electroencephalography and Clinical Neurophysiology 108, no. 2: 123–130. 10.1016/S0168-5597(97)00078-6. 9566625

[psyp70341-bib-0030] Klimesch, W. , B. Schack , and P. Sauseng . 2005. “The Functional Significance of Theta and Upper Alpha Oscillations.” Experimental Psychology 52, no. 2: 99–108. 10.1027/1618-3169.52.2.99. 15850157

[psyp70341-bib-0031] Knoblich, G. , S. Butterfill , and N. Sebanz . 2011. “Psychological Research on Joint Action.” In Psychology of Learning and Motivation. Advances in Research and Theory, edited by B. H. Ross , vol. 54, 59–101. Elsevier. 10.1016/B978-0-12-385527-5.00003-6.

[psyp70341-bib-0032] Krigolson, O. E. , C. D. Hassall , J. Satel , and R. M. Klein . 2015. “The Impact of Cognitive Load on Reward Evaluation.” Brain Research 1627: 225–232. 10.1016/j.brainres.2015.09.028. 26431993

[psyp70341-bib-0033] Krigolson, O. E. , H. Heinekey , C. M. Kent , and T. C. Handy . 2012. “Cognitive Load Impacts Error Evaluation Within Medial‐Frontal Cortex.” Brain Research 1430: 62–67. 10.1016/j.brainres.2011.10.028. 22099261

[psyp70341-bib-0034] Lakens, D. 2013. “Calculating and Reporting Effect Sizes to Facilitate Cumulative Science: A Practical Primer for t‐Tests and ANOVAs.” Frontiers in Psychology 4: 863. 10.3389/fpsyg.2013.00863. 24324449 PMC3840331

[psyp70341-bib-0035] Lakens, D. 2022. “Improving Your Statistical Inferences.” https://lakens.github.io/statistical_inferences/.

[psyp70341-bib-0036] Leckey, M. , and K. D. Federmeier . 2020. “The P3b and P600(s): Positive Contributions to Language Comprehension.” Psychophysiology 57, no. 7: e13351. 10.1111/psyp.13351. 30802979 PMC7934419

[psyp70341-bib-0037] Lee, D. , H. Seo , and M. W. Jung . 2012. “Neural Basis of Reinforcement Learning and Decision Making.” Annual Review of Neuroscience 35: 287–308. 10.1146/annurev-neuro-062111-150512. PMC3490621 22462543

[psyp70341-bib-0038] Lenth, R. V. 2022. “emmeans: Estimated Marginal Means, Aka Least‐Squares Means [Computer Software].” https://CRAN.R‐project.org/package=emmeans.

[psyp70341-bib-0039] Levinson, S. C. 2006. “On the Human ‘Interaction Engine’.” In Roots of Human Sociality, edited by N. J. Enfield and S. C. Levinson , 39–69. Routledge. 10.4324/9781003135517-3.

[psyp70341-bib-0040] Marco‐Pallarés, J. , D. Cucurell , T. Cunillera , et al. 2008. “Human Oscillatory Activity Associated to Reward Processing in a Gambling Task.” Neuropsychologia 46, no. 1: 241–248. 10.1016/j.neuropsychologia.2007.07.016. 17804025

[psyp70341-bib-0077] Maris, E. , and R. Oostenveld . 2007. “Nonparametric Statistical Testing of EEG‐ and MEG‐Data.” Journal of Neuroscience Methods 164, no. 1: 177–190. 10.1016/j.jneumeth.2007.03.024. 17517438

[psyp70341-bib-0041] Meadows, C. C. , P. A. Gable , K. R. Lohse , and M. W. Miller . 2016. “The Effects of Reward Magnitude on Reward Processing: An Averaged and Single Trial Event‐Related Potential Study.” Biological Psychology 118: 154–160. 10.1016/j.biopsycho.2016.06.002. 27288743

[psyp70341-bib-0042] Michelmann, S. , B. Griffiths , and S. Hanslmayr . 2022. “The Role of Alpha and Beta Oscillations in the Human EEG During Perception and Memory Processes.” In The Oxford Handbook of EEG Frequency, edited by P. A. Gable , M. W. Miller , and E. M. Bernat , 202–219. Oxford University Press. 10.1093/oxfordhb/9780192898340.013.10.

[psyp70341-bib-0043] Münte, T. F. , T. P. Urbach , E. Duzel , and M. Kutas . 2000. “Event‐Related Brain Potentials in the Study of Human Cognition and Neuropsychology.” In Handbook of Neuropsychology, edited by F. Boller and J. Grafman , 2nd ed. Elsevier.

[psyp70341-bib-0044] Nassar, M. R. , R. Bruckner , and M. J. Frank . 2019. “Statistical Context Dictates the Relationship Between Feedback‐Related EEG Signals and Learning.” eLife 8: e46975. 10.7554/eLife.46975. 31433294 PMC6716947

[psyp70341-bib-0045] Oostenveld, R. , P. Fries , E. Maris , and J.‐M. Schoffelen . 2011. “Fieldtrip: Open Source Software for Advanced Analysis of MEG, EEG, and Invasive Electrophysiological Data.” Computational Intelligence and Neuroscience 2011: 156869. 10.1155/2011/156869. 21253357 PMC3021840

[psyp70341-bib-0046] Paller, K. A. , and M. Kutas . 1992. “Brain Potentials During Memory Retrieval Provide Neurophysiological Support for the Distinction Between Conscious Recollection and Priming.” Journal of Cognitive Neuroscience 4, no. 4: 375–392. 10.1162/jocn.1992.4.4.375. 23968130

[psyp70341-bib-0047] Perinbanayagam, R. S. 2006. Games and Sport in Everyday Life: Dialogues and Narratives of the Self. Taylor and Francis.

[psyp70341-bib-0048] Peyk, P. , A. De Cesarei , and M. Junghöfer . 2011. “Electromagnetoencephalography Software: Overview and Integration With Other EEG/MEG Toolboxes.” Computational Intelligence and Neuroscience 2011: 861705. 10.1155/2011/861705. 21577273 PMC3090751

[psyp70341-bib-0049] Pfabigan, D. M. , U. Sailer , and C. Lamm . 2015. “Size Does Matter! Perceptual Stimulus Properties Affect Event‐Related Potentials During Feedback Processing.” Psychophysiology 52, no. 9: 1238–1247. 10.1111/psyp.12458. 26059201

[psyp70341-bib-0050] Proudfit, G. H. 2015. “The Reward Positivity: From Basic Research on Reward to a Biomarker for Depression.” Psychophysiology 52, no. 4: 449–459. 10.1111/psyp.12370. 25327938

[psyp70341-bib-0051] R Core Team . 2022. “R: A Language and Environment for Statistical Computing [Computer Software].” Vienna, Austria. https://www.R‐project.org/.

[psyp70341-bib-0052] Rilling, J. K. , and A. G. Sanfey . 2011. “The Neuroscience of Social Decision‐Making.” Annual Review of Psychology 62: 23–48. 10.1146/annurev.psych.121208.131647. 20822437

[psyp70341-bib-0053] Rossion, B. , and G. Pourtois . 2004. “Revisiting Snodgrass and Vanderwart's Object Pictorial Set: The Role of Surface Detail in Basic‐Level Object Recognition.” Perception 33, no. 2: 217–236. 10.1068/p5117. 15109163

[psyp70341-bib-0054] Sambrook, T. D. , and J. Goslin . 2015. “A Neural Reward Prediction Error Revealed by a Meta‐Analysis of ERPs Using Great Grand Averages.” Psychological Bulletin 141, no. 1: 213–235. 10.1037/bul0000006. 25495239

[psyp70341-bib-0055] San Martín, R. 2012. “Event‐Related Potential Studies of Outcome Processing and Feedback‐Guided Learning.” Frontiers in Human Neuroscience 6: 304. 10.3389/fnhum.2012.00304. 23162451 PMC3491353

[psyp70341-bib-0056] Sassenhagen, J. , and I. Bornkessel‐Schlesewsky . 2015. “The P600 as a Correlate of Ventral Attention Network Reorientation.” Cortex 66: A3–A20. 10.1016/j.cortex.2014.12.019. 25791606

[psyp70341-bib-0057] Sassenhagen, J. , and D. Draschkow . 2019. “Cluster‐Based Permutation Tests of MEG/EEG Data Do Not Establish Significance of Effect Latency or Location.” Psychophysiology 56, no. 6: e13335. 10.1111/psyp.13335. 30657176

[psyp70341-bib-0058] Schilbach, L. , B. Timmermans , V. Reddy , et al. 2013. “Toward a Second‐Person Neuroscience.” Behavioral and Brain Sciences 36, no. 4: 393–414. 10.1017/S0140525X12000660. 23883742

[psyp70341-bib-0059] Schupp, H. T. , T. Flaisch , J. Stockburger , and M. Junghöfer . 2006. “Emotion and Attention: Event‐Related Brain Potential Studies.” In Progress in Brain Research: Vol. 156. Understanding Emotions, edited by S. Anders , G. Ende , M. Junghöfer , J. Kissler , and D. Wilgruber , 31–51. Elsevier Science. 10.1016/S0079-6123(06)56002-9. 17015073

[psyp70341-bib-0060] Searle, J. R. 1995. The Construction of Social Reality. Free Press.

[psyp70341-bib-0061] Shultz, S. , and R. I. M. Dunbar . 2012. “The Social Brain Hypothesis: An Evolutionary Perspective on the Neurobiology of Social Behaviour.” In I Know What You're Thinking: Brain Imaging and Mental Privacy, edited by S. D. Richmond . Oxford University Press. 10.1093/acprof:oso/9780199596492.003.0002.

[psyp70341-bib-0062] Sidman, M. 1960. Tactics of Scientific Research. Basic Books.

[psyp70341-bib-0063] Singmann, H. , B. Bolker , J. Westfall , F. Aust , and M. S. Ben‐Shachar . 2021. “afex: Analysis of Factorial Experiments [Computer Software].” https://CRAN.R‐project.org/package=afex.

[psyp70341-bib-0064] Snodgrass, J. G. , and M. Vanderwart . 1980. “A Standardized Set of 260 Pictures: Norms for Name Agreement, Image Agreement, Familiarity, and Visual Complexity.” Journal of Experimental Psychology: Human Learning and Memory 6, no. 2: 174–215. 10.1037//0278-7393.6.2.174. 7373248

[psyp70341-bib-0065] Tomasello, M. 2019. Becoming Human: A Theory of Ontogeny. Belknap Press of Harvard University Press.

[psyp70341-bib-0066] Tomasello, M. , M. Carpenter , J. Call , T. Behne , and H. Moll . 2005. “Understanding and Sharing Intentions: The Origins of Cultural Cognition.” Behavioral and Brain Sciences 28, no. 5: 675–691. 10.1017/S0140525X05000129. 16262930

[psyp70341-bib-0067] Tomlin, D. , M. A. Kayali , B. King‐Casas , et al. 2006. “Agent‐Specific Responses in the Cingulate Cortex During Economic Exchanges.” Science 312, no. 5776: 1047–1050. 10.1126/science.1125596. 16709783

[psyp70341-bib-0068] Vesper, C. , R. P. R. D. van der Wel , G. Knoblich , and N. Sebanz . 2011. “Making Oneself Predictable: Reduced Temporal Variability Facilitates Joint Action Coordination.” Experimental Brain Research 211, no. 3–4: 517–530. 10.1007/s00221-011-2706-z. 21556820 PMC3102185

[psyp70341-bib-0069] Walsh, M. M. , and J. R. Anderson . 2012. “Learning From Experience: Event‐Related Potential Correlates of Reward Processing, Neural Adaptation, and Behavioral Choice.” Neuroscience and Biobehavioral Reviews 36, no. 8: 1870–1884. 10.1016/j.neubiorev.2012.05.008. 22683741 PMC3432149

[psyp70341-bib-0070] Wang, C. , and Q. Zhang . 2021. “Word Frequency Effect in Written Production: Evidence From ERPs and Neural Oscillations.” Psychophysiology 58, no. 5: e13775. 10.1111/psyp.13775. 33522614

[psyp70341-bib-0071] Wickham, H. 2016. ggplot2: Elegant Graphics for Data Analysis. Springer‐Verlag.

[psyp70341-bib-0072] Wickham, H. , M. Averick , J. Bryan , et al. 2019. “Welcome to the Tidyverse.” Journal of Open Source Software 4, no. 43: 1686. 10.21105/joss.01686.

[psyp70341-bib-0073] Williams, C. C. , T. D. Ferguson , C. D. Hassall , W. Abimbola , and O. E. Krigolson . 2021. “The ERP, Frequency, and Time‐Frequency Correlates of Feedback Processing: Insights From a Large Sample Study.” Psychophysiology 58, no. 2: e13722. 10.1111/psyp.13722. 33169842

[psyp70341-bib-0074] Wurm, F. , W. Walentowska , B. Ernst , M. C. Severo , G. Pourtois , and M. Steinhauser . 2021. “Task Learnability Modulates Surprise but Not Valence Processing for Reinforcement Learning in Probabilistic Choice Tasks.” Journal of Cognitive Neuroscience 34, no. 1: 34–53. 10.1162/jocn_a_01777. 34879392

[psyp70341-bib-0075] Yeung, N. , and A. G. Sanfey . 2004. “Independent Coding of Reward Magnitude and Valence in the Human Brain.” Journal of Neuroscience 24, no. 28: 6258–6264. 10.1523/jneurosci.4537-03.2004. 15254080 PMC6729539

[psyp70341-bib-0076] Yoshida, W. , B. Seymour , K. J. Friston , and R. J. Dolan . 2010. “Neural Mechanisms of Belief Inference During Cooperative Games.” Journal of Neuroscience 30, no. 32: 10744–10751. 10.1523/jneurosci.5895-09.2010. 20702705 PMC2967416

