# Supplementary material for: The Brain Signature of Reward Processing During Cooperative Gaming
Source: Psychophysiology. 2026 Jul 28;63(7):e70341. doi: 10.1111/psyp.70341 (PMC13413252; doi:10.1111/psyp.70341)
Supplement: Supplementary file 1 — Figure S1: Illustration of sender‐receiver role differences in induced oscillations (1–40 Hz) during SR game trials for selecting Receivers (Picture 1) and monitoring Receivers (Picture 2), respectively. Sender‐receiver role differences in induced oscillations were traced for selecting Receivers (a) during presentation of the first picture cue (1000 ms) and (b) for a prolonged time spanning the fixation cross (500 ms) and the first second of the picture selection array (500–1400 ms), using the same baseline interval from 200 to 100 ms before onset of Picture 1. (c) A similar effect was found during the presentation of the second picture cue (1000 ms). Left. Time‐frequency plots of t‐values illustrate the significant clusters aggregated over sensors. The summed cluster t‐value at each time × frequency point is standardized by dividing it by the number of significant sensors within the respective cluster. Data points not significant at cluster level are masked. Middle. The topography of the cluster is shown in the display of the sensor layout (nose pointing up) with the size of the marked sensors being proportional to their contribution to the cluster. For more details about the spatial extension of the frequency clusters please refer to Figures S5–S7. Right. Violin plots show individual cluster averages expressed as power change (to baseline) in decibel across time, frequency, and sensors as a function of role. Cluster averages of each subject are weighted within time, frequency, and sensor dimensions. Paired measures of all 48 participants are traced by gray lines when exhibiting the direction of effect observed by the cluster‐based statistic (see left) and orange lines when exhibiting a reversed pattern. Colored center lines within box plots represent the mean, edges mark the 25th and 75th percentiles, whiskers denote 1.5 times the interquartile range. Figure S2: Illustration of sender‐receiver role differences in induced oscillations (1–40 Hz) during SR_noPR/SR_PR [file PSYP-63-e70341-s001.pdf]

SUPPLEMENT

To

The Brain Signature of Reward Processing during Cooperative Gaming

Karl-Philipp Flösch[1,2], Tobias Flaisch[1], Marco Steinhauser[3], Harald T. Schupp[1,2]

[1] Department of Psychology, University of Konstanz, 78457 Konstanz, Germany

[2] Centre for the Advanced Study of Collective Behaviour, University of Konstanz, 78457 Konstanz, Germany

[3] Department of Psychology, Catholic University of Eichstätt-Ingolstadt, 85072, Eichstätt, Germany

SUPPLEMENTARY ANALYSES

(S-I) Brain oscillations 1-40 Hz: Sender-receiver differences in *SR* and *SR_noPR* trials

***SR* Trials.** *Receivers* of a task-relevant picture cue (*Picture 1*) showed a significant alpha/beta power decrease in comparison to *Senders* (see Fig. S1a). A significant ERD effect (p = .0002; d = -1.15) was observed in a widespread cluster, most pronounced over central and parietal areas and in the alpha band from ~8 to 15 Hz. The cluster effect already begins at stimulus onset, subsides for about 300 ms and reappears at ~400 to 500 ms. Notably, Figure S1b indicates that receiver-sender differences persisted beyond the offset of picture presentation during the fixation cross (0 to 500 ms) as well as during the first second of the selection array (500 to 1400 ms) with similar characteristics in topography and frequency space (p = .0002; d = -1.05).

Furthermore, receiving a cue that was informative about the partner's decision (*Picture 2*) elicited a significant ERD cluster compared with *Senders* (p = .0002; d = -0.93; see Fig. S1c) most pronounced over central and parietal regions and within the alpha band (~9-14 Hz). The temporal characteristics were very similar to the alpha/beta ERD observed for *Picture 1*.

No significant negative clusters were found when contrasting selecting with monitoring *Receivers* (ps ≥ .16) or *Senders* (ps ≥ .21).

***SR_noPR* Trials.** Again, selecting *Receivers* (*Picture 1*) exhibited a significant alpha to beta ERD (p = .0002; d = -1.05; see Fig. S2a), most pronounced over centro-parietal regions and in a frequency range of ~8 to 15 Hz, which begins at stimulus onset, subsides for about 200 ms and reappears at ~300 to 400 ms. This effect persisted during the fixation period as well as the first second of the selection array (p = .0002; d = -0.98; see Fig. S2b). Furthermore, monitoring *Receivers* having the chance to win a bonus but did not receive it (*SR_noPR*) as well as those obtaining a personal reward (*SR_PR*) showed significant negative cluster effects within the alpha to lower beta range compared to *Senders* (ps = .0002; ds = -0.99 and -1.09; see Fig. S2c and S2d) with temporal characteristics similar to *Picture 1* but with a slightly more occipital topography.

No significant negative or positive clusters were found when contrasting selecting with monitoring *Receivers* (ps ≥ .08) or *Senders* (ps ≥ .31).

Brain oscillations 1-40 Hz: Selecting vs. monitoring receiver

A significant ERD effect (~10-25 Hz) was observed from 300 to 850 ms, most pronounced in in the upper beta band (~20-25 Hz) when contrasting selecting with monitoring *Receivers* (p = .012; d = -0.64; see Fig. S10). However, this cluster did not meet the prespecified significance criterion of $p < .005$.

No further negative clusters were significant when comparing within *Receiver* roles (selecting vs. monitoring; ps ≥ .08) or within *Sender* roles (ps ≥ .31) roles.

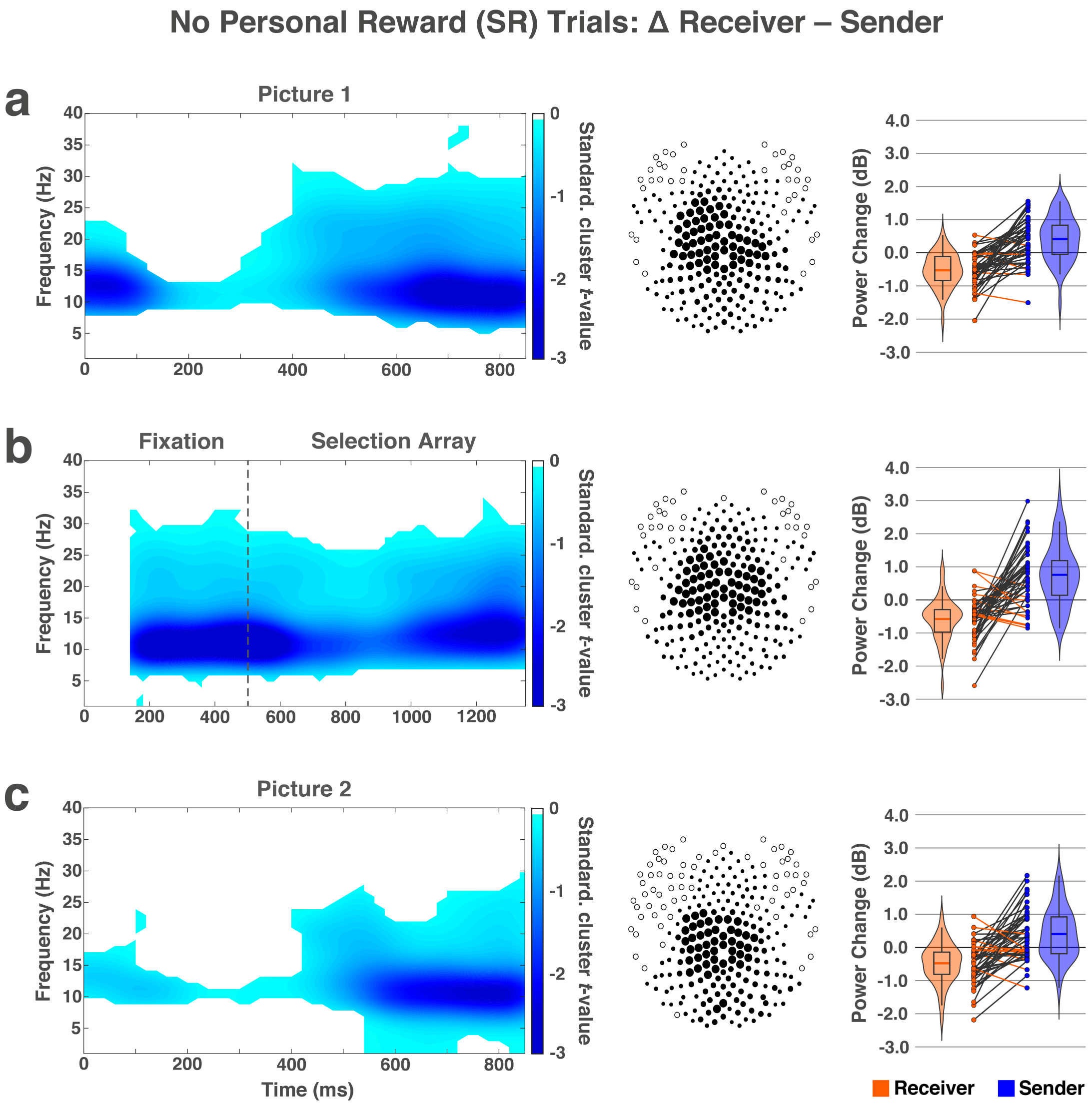

Fig. S1. Illustration of sender-receiver role differences in induced oscillations (1-40 Hz) during *SR* game trials for selecting *Receivers* (*Picture 1*) and monitoring *Receivers* (*Picture 2*), respectively.

Sender-receiver role differences in induced oscillations were traced for selecting *Receivers* **(a)** during presentation of the first picture cue (1000 ms) and **(b)** for a prolonged time spanning the fixation cross (500 ms) and the first second of the picture selection array (500-1400 ms), using the same baseline interval from 200 to 100 ms before onset of *Picture 1*. **(c)** A similar effect was found during the presentation of the second picture cue (1000 ms).

Left. Time-frequency plots of *t*-values illustrate the significant clusters aggregated over sensors. The summed cluster *t*-value at each time x frequency point is standardized by dividing it by the number of significant sensors within the respective cluster. Data points not significant at cluster level are masked.

Middle. The topography of the cluster is shown in the display of the sensor layout (nose pointing up) with the size of the marked sensors being proportional to their contribution to the cluster. For more details about the spatial extension of the frequency clusters please refer to Fig. S5-S7.

Right. Violin plots show individual cluster averages expressed as power change (to baseline) in decibel across time, frequency, and sensors as a function of role. Cluster averages of each subject are weighted within time, frequency, and sensor dimensions. Paired measures of all 48 participants are traced by gray lines when exhibiting the direction of effect observed by the cluster-based statistic (see left) and orange lines when exhibiting a reversed pattern. Colored center lines within box plots represent the mean, edges mark the 25th and 75th percentiles, whiskers denote 1.5 times the interquartile range.

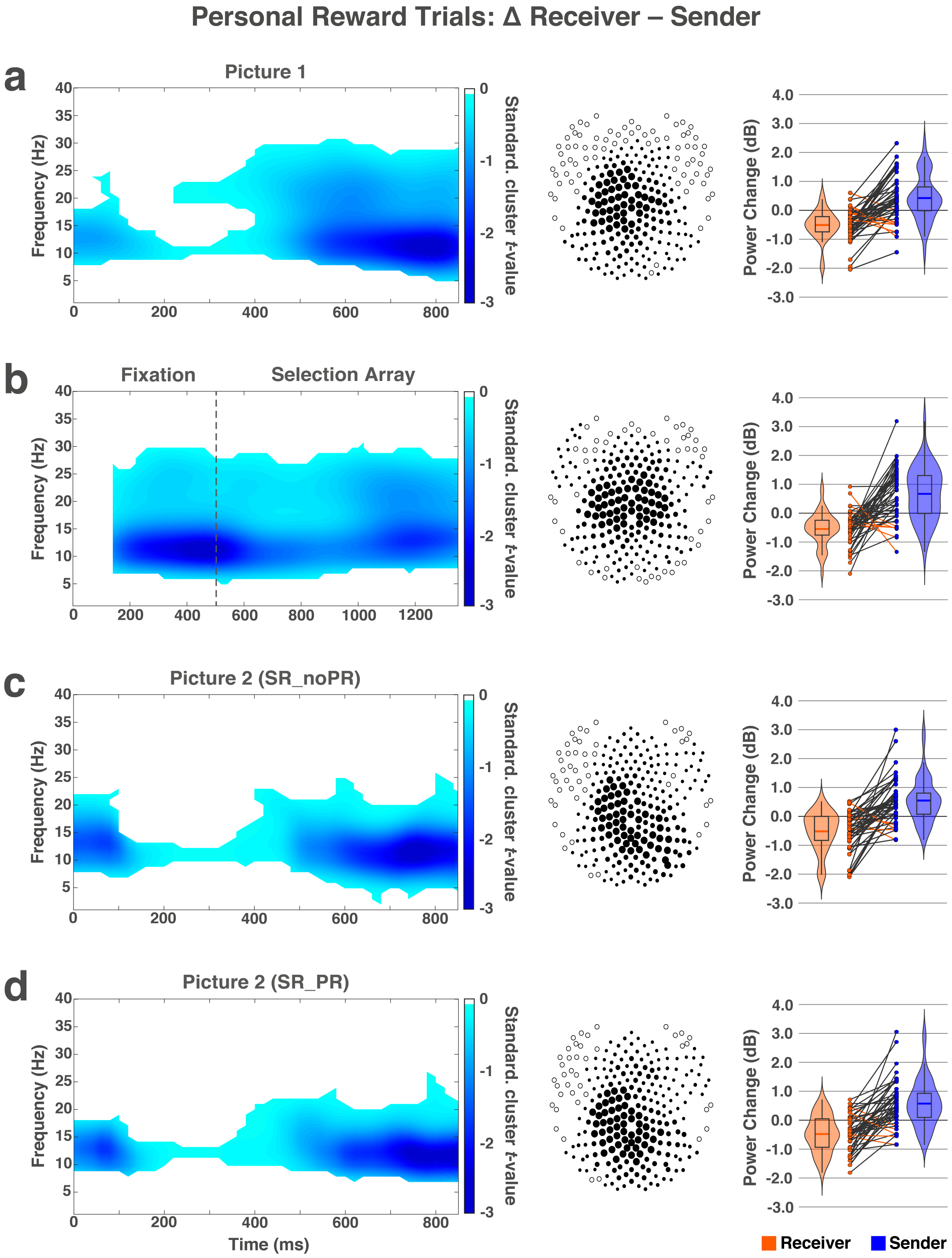

Fig. S2. Illustration of sender-receiver role differences in induced oscillations (1-40 Hz) during *SR_noPR/SR_PR* game trials for selecting *Receivers* (*Picture 1*) and monitoring *Receivers* (*Picture 2*), respectively.
Sender-receiver role differences in induced oscillations were traced for selecting *Receivers* **(a)** during presentation of the first picture cue (1000 ms) and **(b)** for a prolonged time spanning the fixation cross (500 ms) and the first second of the picture selection array (500-1400 ms), using the same baseline interval from 200 to 100 ms before onset of *Picture 1*. **(c+d)** A similar effect was found during the presentation of the second picture cue (1000 ms).
For a description of the different panels, please refer to Fig. S1. For more details about the spatial extension of the frequency clusters please refer to Fig. S5-S8.

(S-II) Brain oscillations 1-40 Hz: Late vs. early sender-receiver differences

Exploratory testing revealed that the early ERD effects (*Receiver* vs. *Sender*; see main analysis) from ~0 to 200 ms seem to be driven by the sender role, while the later effects from ~500 to 850 ms are mainly driven by the receiver role (all t-tests one-tailed, uncorrected). That is, *Senders* show a stronger alpha/beta increase compared to baseline in an early time window from 0 to 200 ms (*Picture 1*: $M = 0.25$ dB, $t(47) = 5.88$, $p < .0001$; *Picture 2*: $M = 0.30$ dB, $t(47) = 4.93$, $p < .0001$) than in a late time window from 500 to 850 ms (*Picture 1*: $M = 0.15$ dB, $t(47) = 2.61$, $p = .006$; *Picture 2*: $M = 0.12$ dB, $t(47) = 2.03$, $p = .024$), ts(47) ≥ 1.62, ps ≤ .056, ds ≥ 0.23; Δ ≥ 0.11 dB. *Receivers*, on the other hand, exhibit a larger alpha/beta decrease compared to baseline in the late (*Picture 1*: $M = -0.30$ dB, $t(47) = -7.65$, $p < .0002$; *Picture 2*: $M = -0.32$ dB, $t(47) = 6.29$, $p < .0001$) as compared to the early time window (*Picture 1*: $M = -0.13$ dB, $t(47) = -3.76$, $p = .0002$; *Picture 2*: $M = -0.15$ dB, $t(47) = -2.59$, $p = .005$), ts(47) ≤ -2.63, ps ≤ .0056, ds ≤ -0.38; Δ ≤ -0.17 dB. This pattern in early ERD effects may stem from motor-related alpha/beta perturbations of *Senders* post response, which was reported previously in the Pacman Game (see Flösch et al. 2024a).

(S-III) Brain oscillations 1-100 Hz: Event-related synchronizations (ERS)

A positive power difference from about 1-40 Hz was observed for *Receivers* compared to *Senders* (*SR*: p = .011 *ns*; d = 0.58; *SR_noPR*: p = .0024 and .008; d = 1.16; see Fig. S11 and S12) mainly beginning around the onset of the selection array (at 1500 ms after onset of *Picture 1*) and lasting for 1 s over posterior sensor sites, most pronounced within the delta to theta band (~1-5 Hz) and the upper beta to gamma bands (~20-40 Hz). In addition, a strong gamma ERS was observed in a similar time window from 40 to 100 Hz, broadly distributed over mainly centro-parietal sensors (*SR*: p = .0002; d = 0.90; *SR_noPR*: p = .0002; d = 1.11; see Fig. S13 and S14). This gamma effect was also found in a previous study (Flösch et al. 2024a) and may indicate perceptual sensitivity for unpredictable stimuli during a visual search and memory matching task (cf. Sauseng et al. 2009; Biel et al. 2021).

Supplementary references not included in the main text:

Biel, A. L., Minarik, T., Sauseng, P. (2021). EEG cross-frequency phase synchronization as an index of memory matching in visual search. *NeuroImage, 235,* 117971, doi:10.1016/j.neuroimage.2021.117971

Sauseng, P., Klimesch, W., Heise, K. F., Gruber, W. R., Holz, E., Karim, A. A., et al. (2009). Brain oscillatory substrates of visual short-term memory capacity. *Current biology: CB, 19,* 1846–52, doi:10.1016/j.cub.2009.08.062.

(S-IV) Event-related Potentials: Sender-receiver differences in ***SR*** and ***SR_noPR*** trials

Table S1

Posterior Negativity (140-200 ms): ANOVA model including the factor Trial Type (SR vs. SR_noPR)

	Source	*df*	MSE	*F*	η_g^2	η_p^2	*p*
1	**Communication**	**1, 47**	**6.32**	**45.51**	**.14**	**.49**	**<.0001**
2	**Picture**	**1, 47**	**0.61**	**11.07**	**.004**	**.19**	**.002**
3	Trial Type	1, 47	0.39	2.52	<.001	.05	.12
4	Communication:Picture	1, 47	1.01	1.10	<.001	.02	.30
5	Communication:Trial Type	1, 47	0.47	0.00	<.001	<.001	.99
6	Picture:Trial Type	1, 47	0.61	0.81	<.001	.02	.37
7	Communication:Picture:Trial Type	1, 47	0.49	2.20	<.001	.04	.14

Please note that the *SR_PR* condition was removed from the cell means for this ANOVA model. Differences related to reward are analyzed in a separate cluster-corrected analysis (see main Results section).

Table S2

Central Positivity (300-600 ms): ANOVA model including the factor Trial Type (SR vs. SR_noPR)

	Source	*df*	MSE	*F*	η_g^2	η_p^2	*p*
1	**Communication**	**1, 47**	**5.45**	**164.19**	**.44**	**.78**	**<.0001**
2	**Picture**	**1, 47**	**1.09**	**11.85**	**.01**	**.20**	**.001**
3	**Time**	**1, 47**	**2.08**	**5.43**	**.010**	**.10**	**.02**
4	Trial Type	1, 47	0.60	0.14	<.001	.003	.71
5	**Communication:Picture**	**1, 47**	**0.87**	**11.63**	**.009**	**.20**	**.001**
6	**Communication:Time**	**1, 47**	**0.87**	**35.01**	**.03**	**.43**	**<.0001**
7	Picture:Time	1, 47	0.13	0.07	<.001	.002	.79
8	Communication:Trial Type	1, 47	0.64	0.64	<.001	.01	.43
9	Picture: Trial Type	1, 47	0.54	0.00	<.001	<.001	.95
10	Time: Trial Type	1, 47	0.13	0.34	<.001	.007	.56
11	Communication:Picture:Time	1, 47	0.14	0.24	<.001	.005	.63
12	Communication:Picture:Trial Type	1, 47	0.70	1.48	<.001	.03	.23
13	Communication:Time:Trial Type	1, 47	0.05	0.60	<.001	.01	.44
14	Picture:Time:Trial Type	1, 47	0.13	0.00	<.001	<.001	.97
15	Communication:Picture:Time: Trial Type	1, 47	0.10	2.80	<.001	.06	.10

Please note that the *SR_PR* condition was removed from the cell means for this ANOVA model. Differences related to reward are analyzed in a separate cluster-corrected analysis (see main Results section).

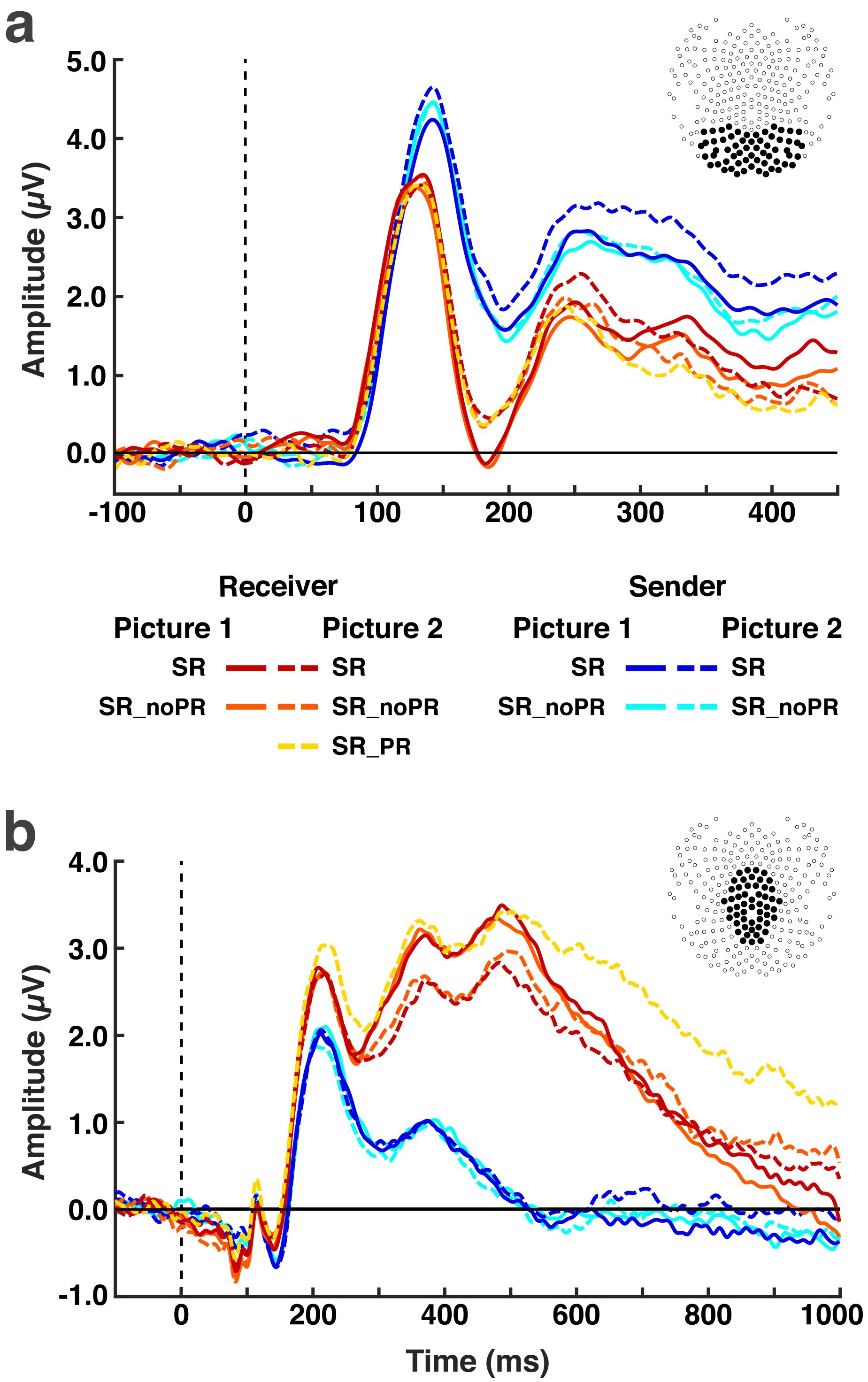

Fig. S3. Grand mean ERP waveforms of *Receivers* and *Senders* in an occipito-temporal (a) and a centro-parietal (b) sensor cluster, respectively.

(a) *Receivers* compared with *Senders* show a relative negative deflection at occipito-temporal sensor sites.

(b) *Receivers* compared with *Senders* show an enhanced P3 component (300-400 ms) and subsequent late positivity (400-600 ms) at centro-parietal sensor sites. *Receivers* obtaining a personal reward (*SR_PR*) exhibit a sustained late positive potential compared to all other player roles (600-1000 ms).

Note. ERP waveforms were calculated using the cluster as displayed in the sensor layouts (nose pointing up), zero-phase lowpass-filtered at 14 Hz for illustrative purposes.

SUPPLEMENTARY FIGURES

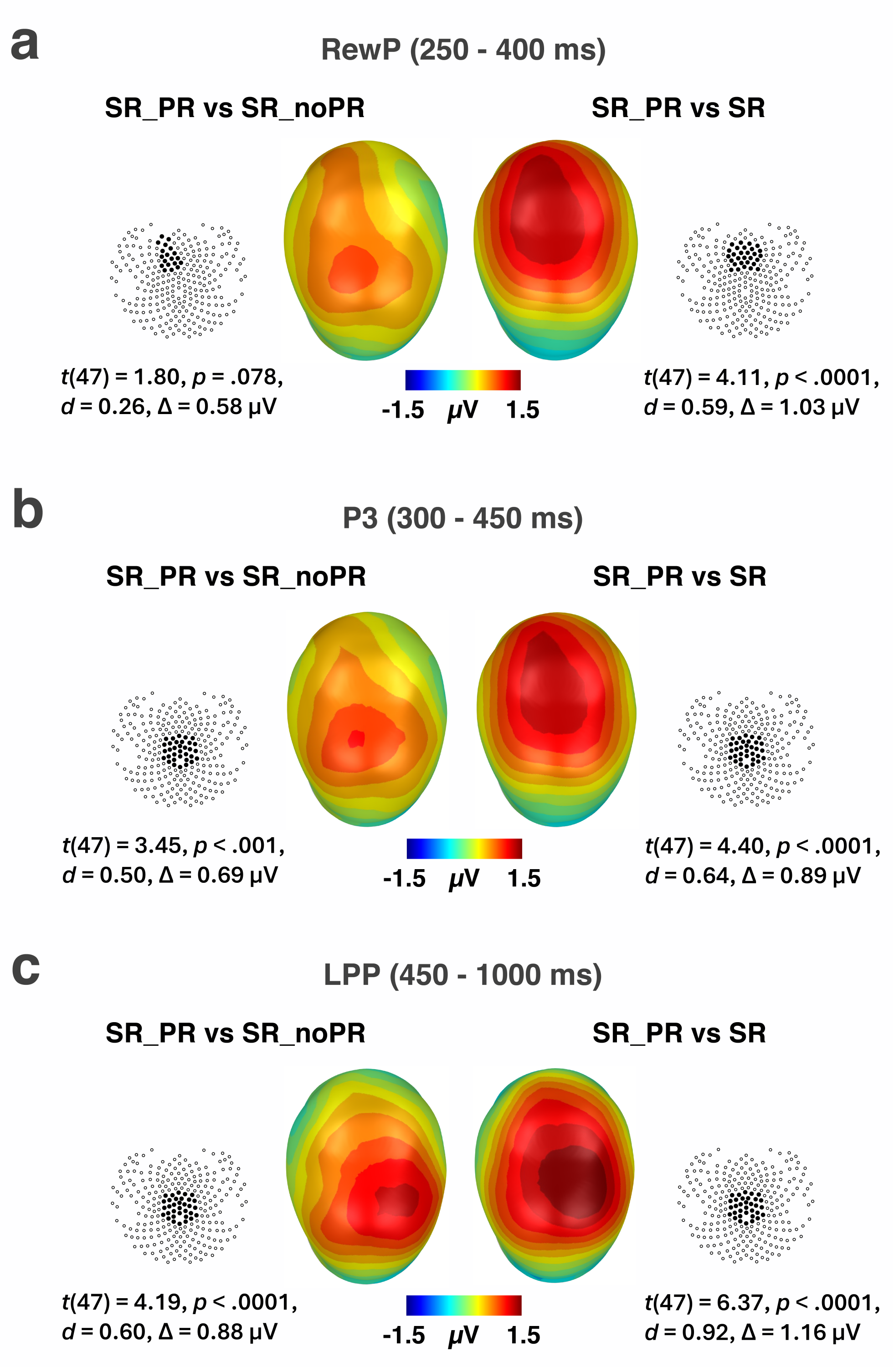

Fig. S4. Illustration of personal reward effects in monitoring receivers associated with (a) the Reward Positivity (250-400 ms), (b) the P3 component (300-450 ms), and (c) the Late Positive Potential (450-1000 ms).
Scalp difference maps in top view (nose pointing up) indicate larger amplitude deflections when monitoring receivers obtained a personal reward (*SR_PR*) compared to trials in which a personal reward was attainable but not received (*SR_noPR*) and trials in which no additional bonus could be obtained (*SR*). Only correct trials were analyzed, ensuring that a shared reward was received in all three trial types.
Note. Sensor layouts depict the clusters used for analysis. All *t*-tests were conducted two-sided, *p*-values are uncorrected, Cohen's *d* was calculated by $t/\sqrt{n}$. In the main Results section, these data were analyzed using a cluster-based permutation approach.

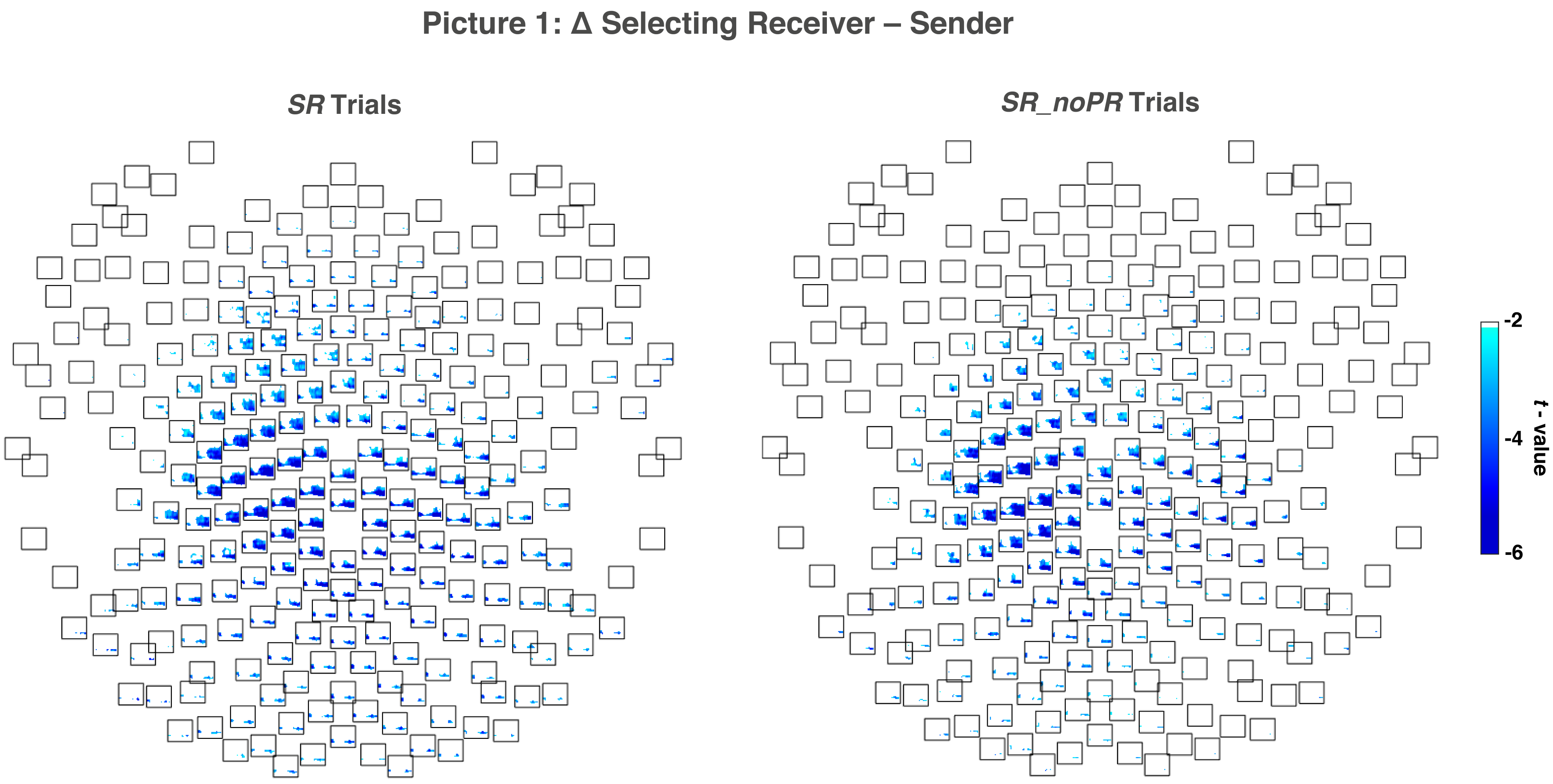

Fig. S5. Illustration of sender-receiver role differences in induced oscillations (1-40 Hz) for *Selecting Receivers* (*Picture 1*) during *SR* and *SR_noPR* trials, respectively. Time-frequency plots of *t*-values illustrate respective clusters (see Fig. S1 and S2) at each sensor site from 0 to 850 ms post stimulus onset and from 1 to 40 Hz. Non-significant data points are masked at an alpha-level of p = .025 (one-tailed).

Fixation/Selection Array: Δ Selecting Receiver – Sender

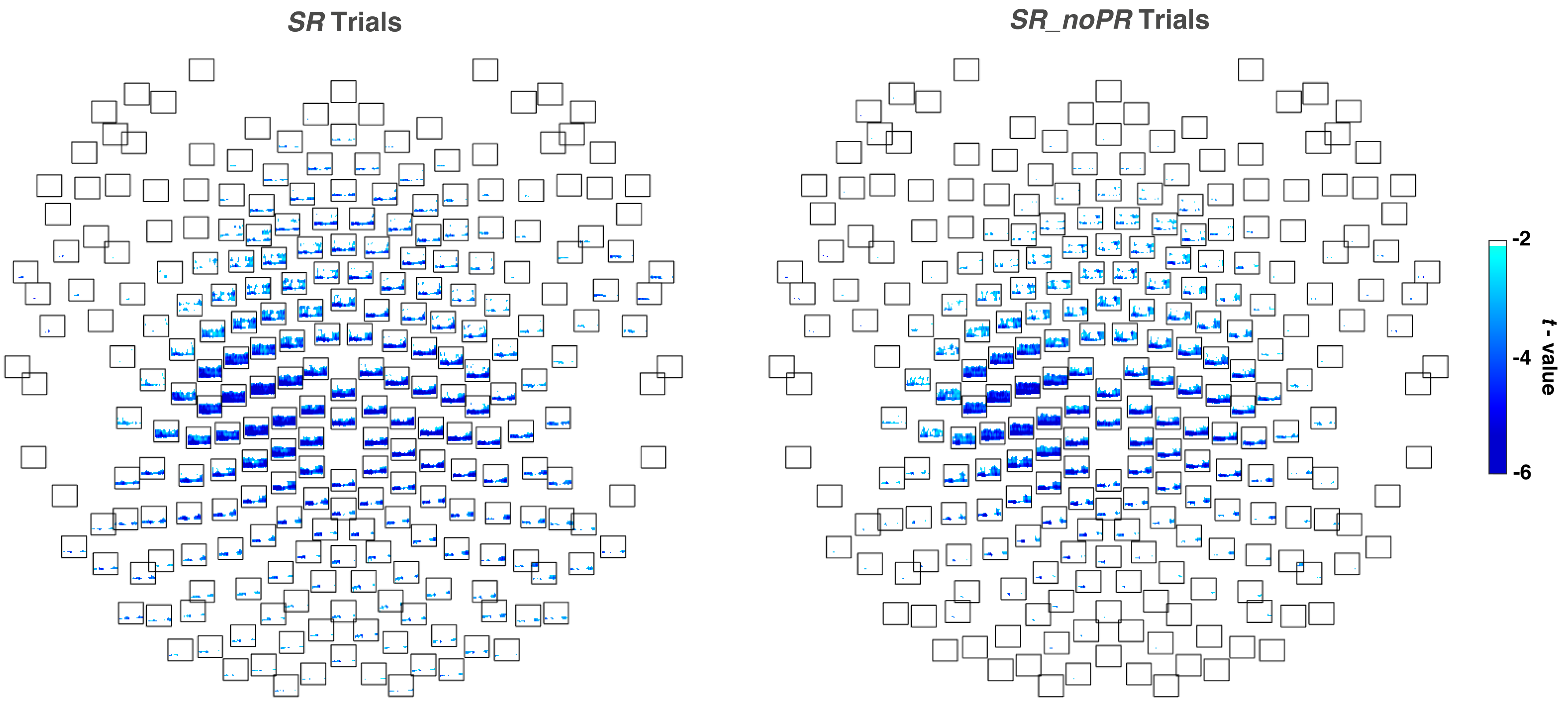

Fig. S6. Illustration of sender-receiver role differences in induced oscillations (1-40 Hz) for *Selecting Receivers* during *SR* and *SR_noPR* trials, respectively.
Time-frequency plots of *t*-values illustrate respective clusters (see Fig. S1 and S2) at each sensor site and from 1 to 40 Hz for a prolonged time spanning the fixation cross (500 ms) and the first second of the picture selection array (500-1400 ms). Non-significant data points are masked at an alpha-level of p = .025 (one-tailed).

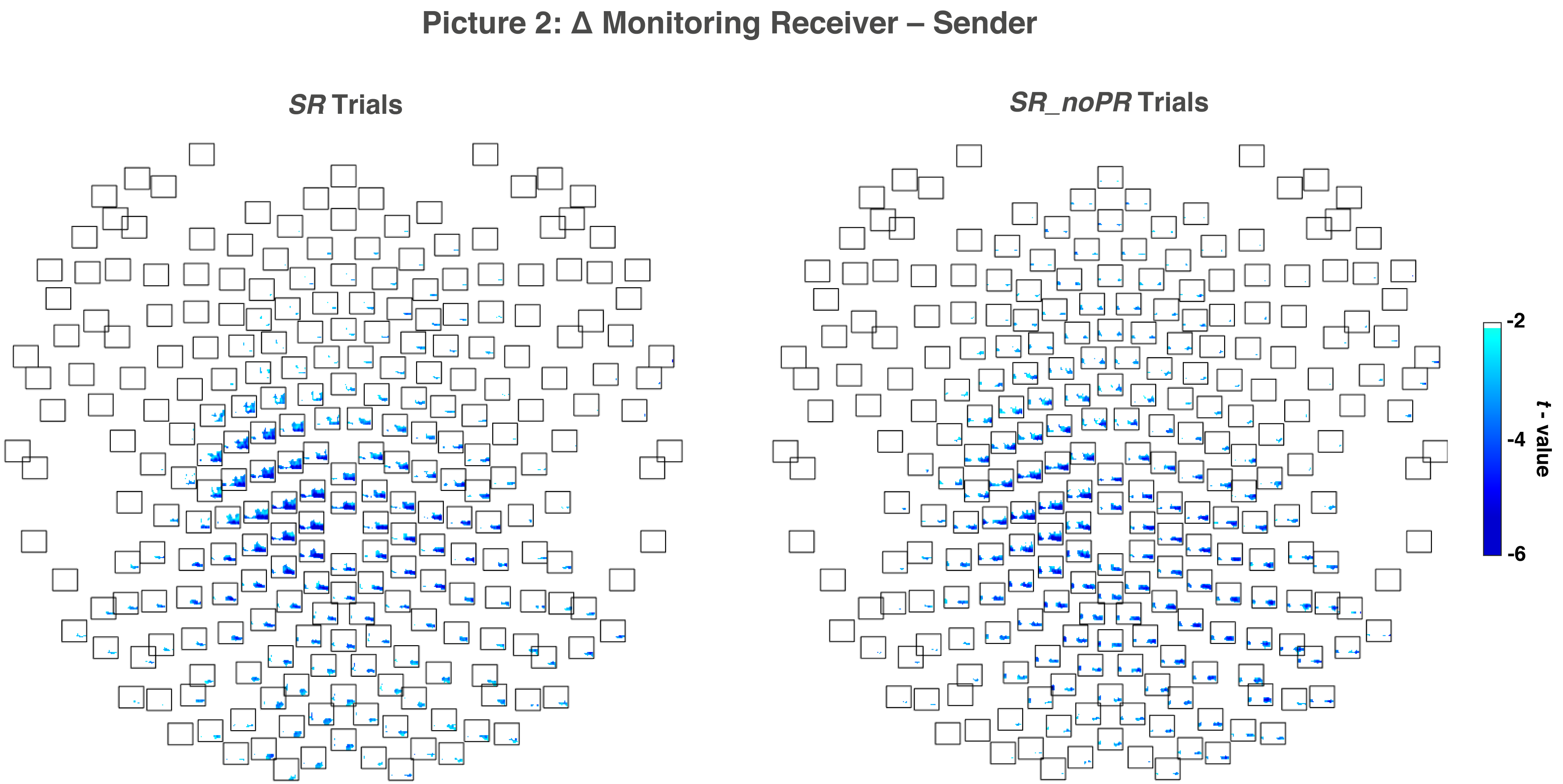

Fig. S7. Illustration of sender-receiver role differences in induced oscillations (1-40 Hz) for *Monitoring Receivers* (*Picture 2*) during *SR* and *SR_noPR* trials, respectively. Time-frequency plots of *t*-values illustrate respective clusters (see Fig. S1 and S2) at each sensor site from 0 to 850 ms post stimulus onset and from 1 to 40 Hz. Non-significant data points are masked at an alpha-level of p = .025 (one-tailed).

Picture 2: Δ Monitoring Receiver – Sender

SR_PR Trials

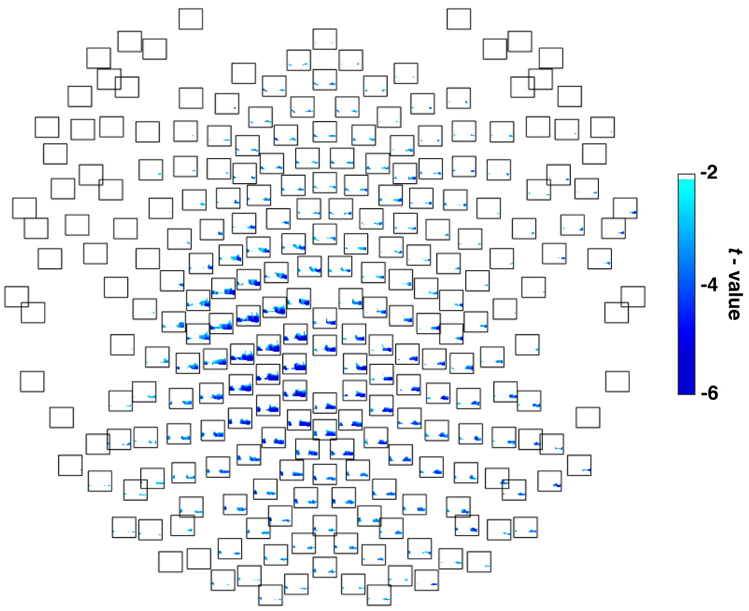

Fig. S8. Illustration of sender-receiver role differences in induced oscillations (1-40 Hz) for *Monitoring Receivers* (*Picture 2*) wo obtained a personal reward (*SR_PR*).

Time-frequency plots of *t*-values illustrate respective clusters (see Fig. S1 and S2) at each sensor site from 0 to 850 ms post stimulus onset and from 1 to 40 Hz. Non-significant data points are masked at an alpha-level of p = .025 (one-tailed).

Reward in Monitoring Receivers (Picture 2)

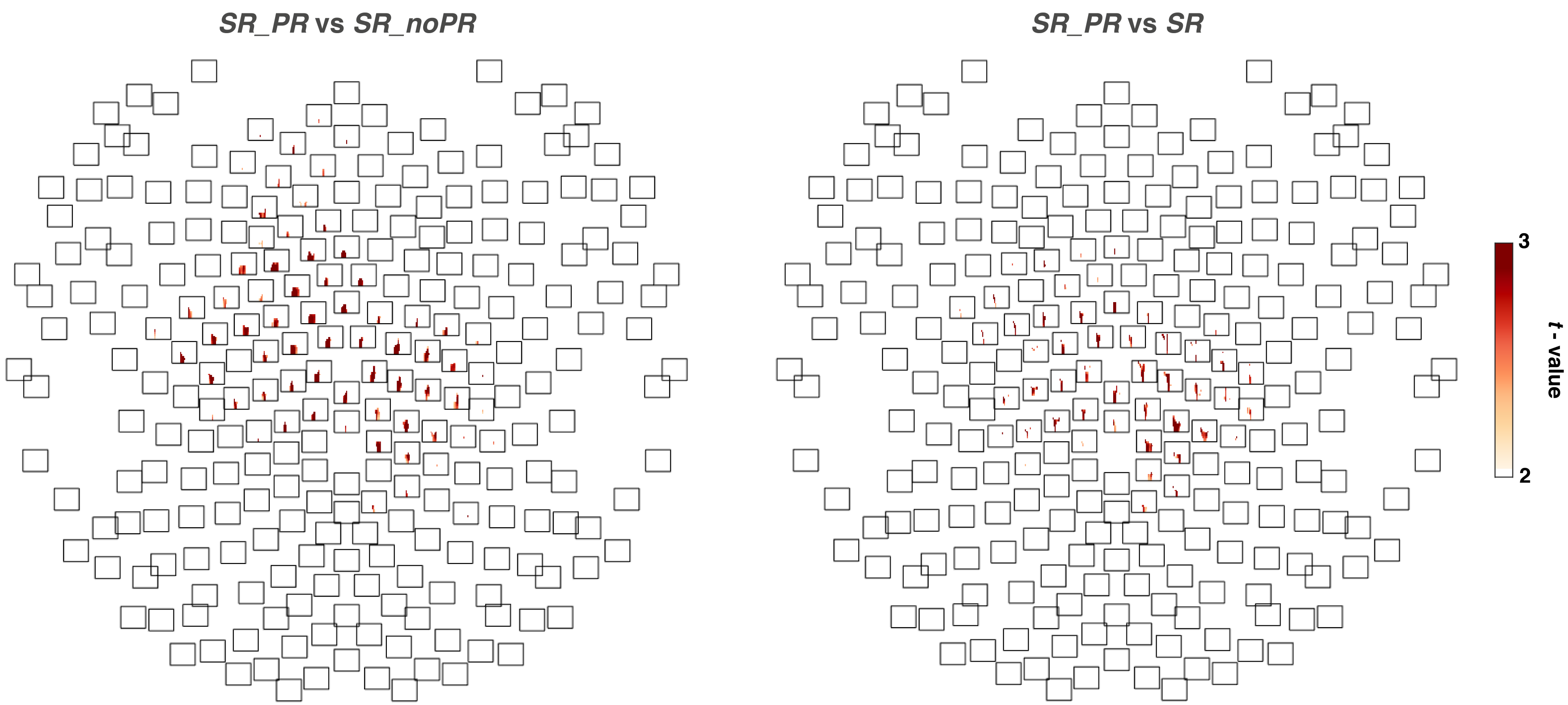

Fig. S9. Illustration of role effects in induced oscillations (1-40 Hz) comparing trials where monitoring *Receivers* (*Picture 2*) actually won a personal reward (*SR_PR*) versus (a) trials where they could obtain but did not receive a personal reward (*SR_noPR*) and (b) trials where they could not obtain an additional bonus (*SR*). Please note that only correct trials were included in the analyses, assuring that a shared reward was received in all three trial types.

Time-frequency plots of *t*-values illustrate respective clusters (see Fig. 2 of main results) at each sensor site from 0 to 850 ms post stimulus onset and from 1 to 40 Hz. Non-significant data points are masked at an alpha-level of p = .025 (one-tailed). The y-axis shows logarithmic frequency in Hz.

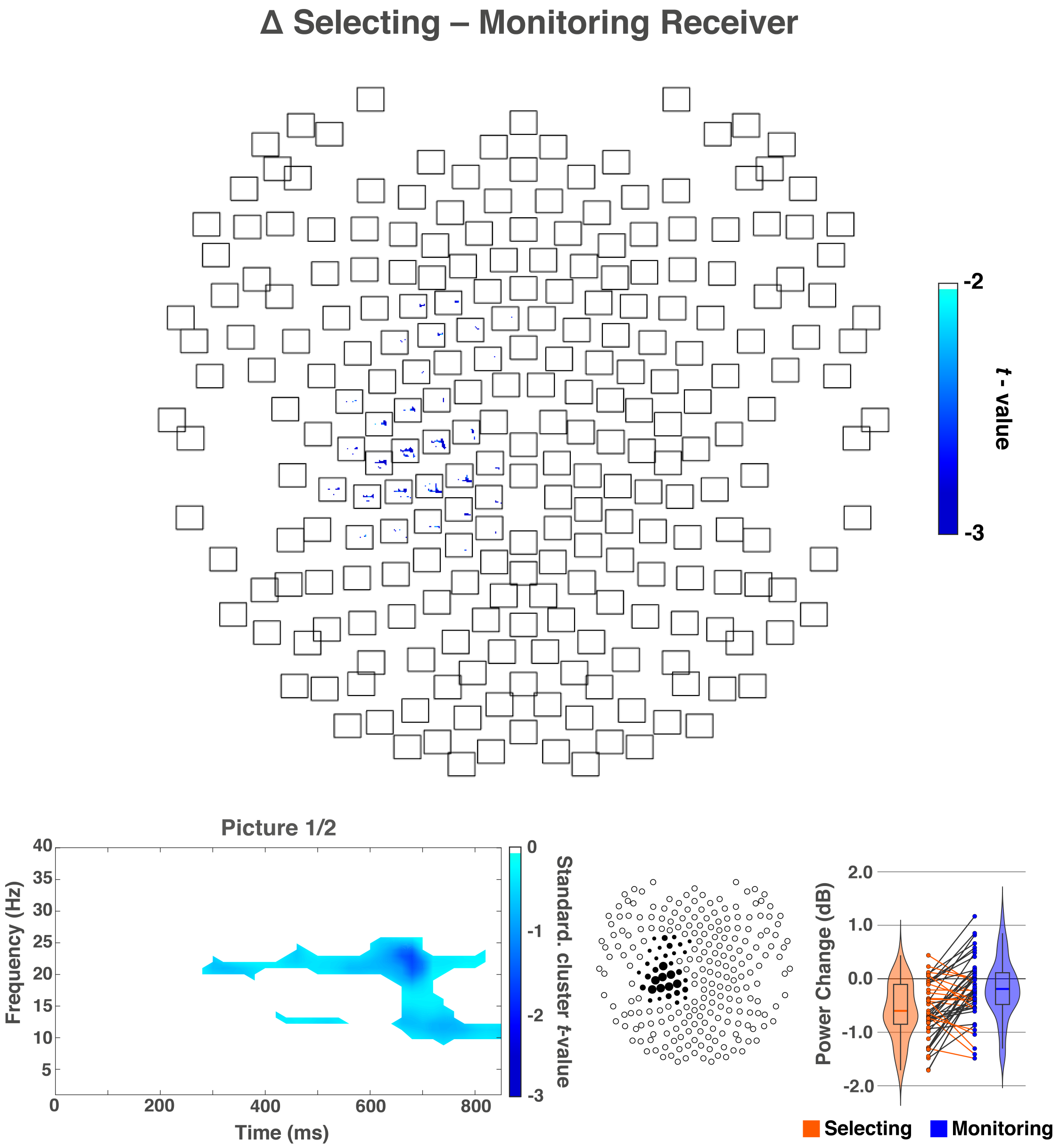

Fig. S10. Illustration of a significant negative cluster (p = .012, d = -0.64) in induced oscillations (1-40 Hz) for *Selecting Receivers* as compared to *Monitoring Receivers*.

The pictures were presented in a time window of 0 to 1000 ms. For a description of the different panels, please refer to Fig. S1 and S5.

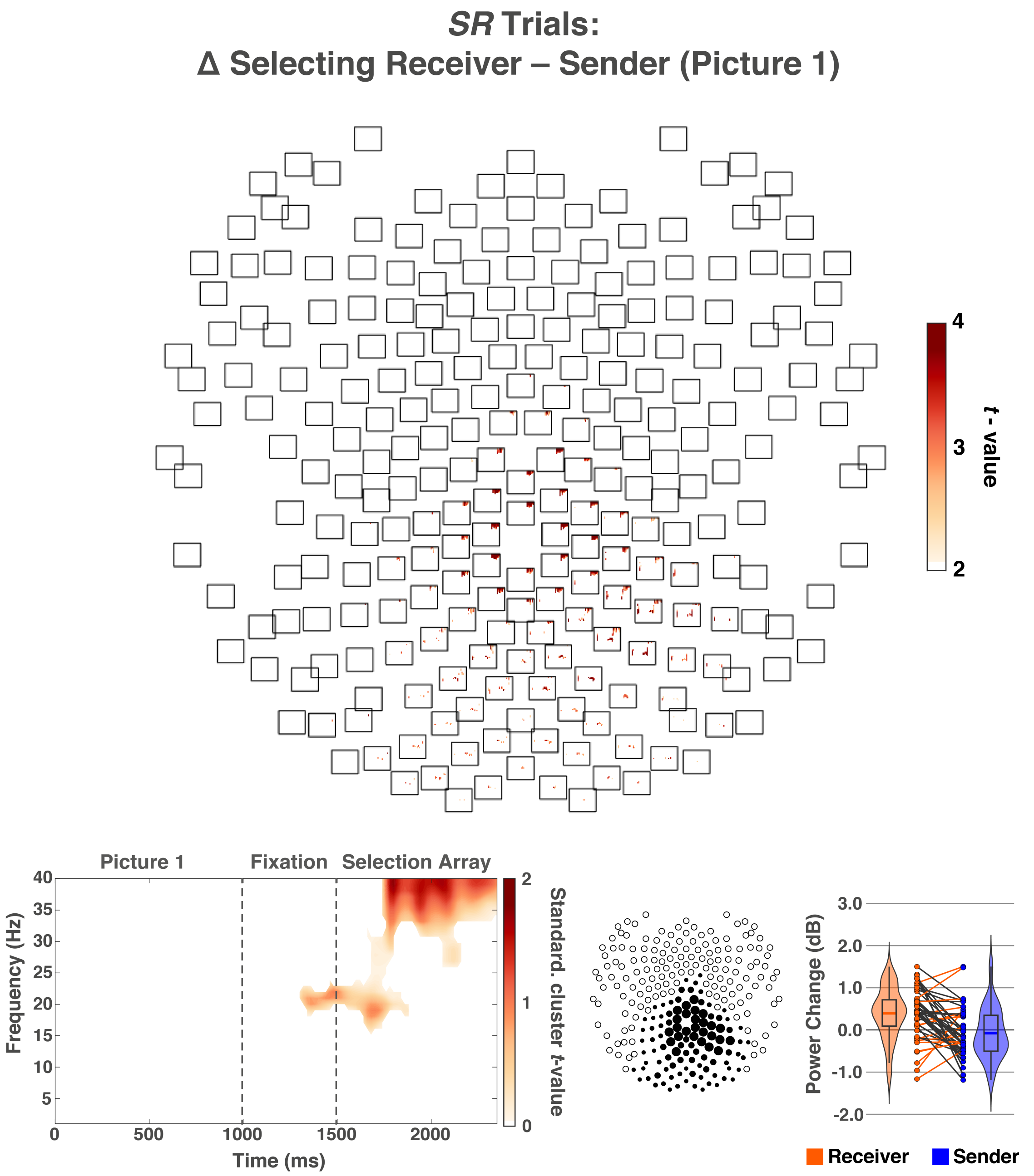

Fig. S11. Illustration of a significant positive cluster (p = .011, d = 0.58) in induced oscillations (1-40 Hz) for *Selecting Receivers* as compared to *Senders* during *SR* trials.

The picture was presented in a time window of 0 to 1000 ms, followed by a fixation cross for 500 ms and the first second of the picture selection array (1500-2500 ms). For a description of the different panels, please refer to Fig. S1 and S5.

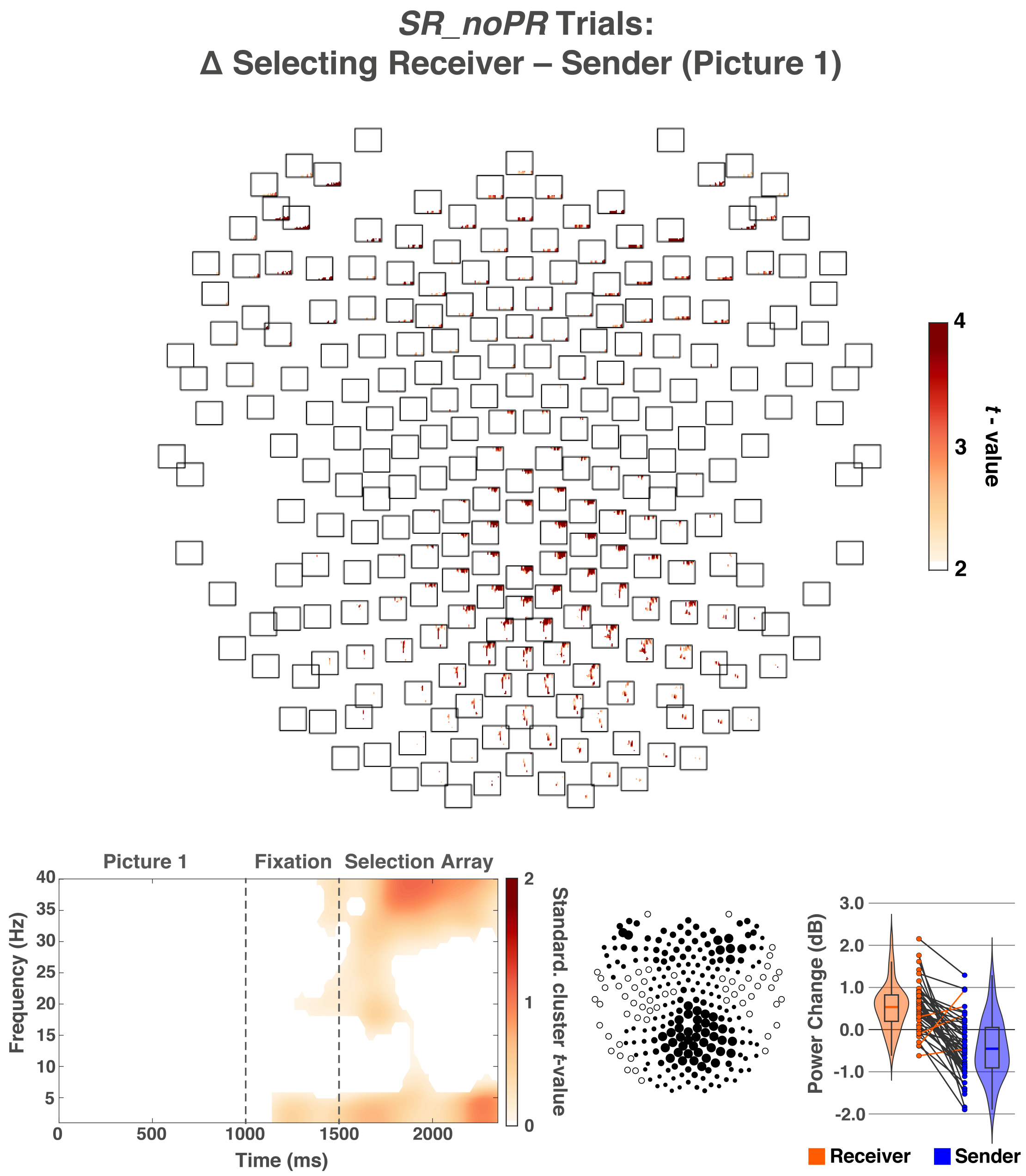

Fig. S12. Illustration of a significant positive cluster (*p = .0024 and .008; d = 1.16*) in induced oscillations (1-40 Hz) for *Selecting Receivers* as compared to *Senders* during *SR_noPR* trials.
The picture was presented in a time window of 0 to 1000 ms, followed by a fixation cross for 500 ms and the first second of the picture selection array (1500-2500 ms). For a description of the different panels, please refer to Fig. S1 and S5.

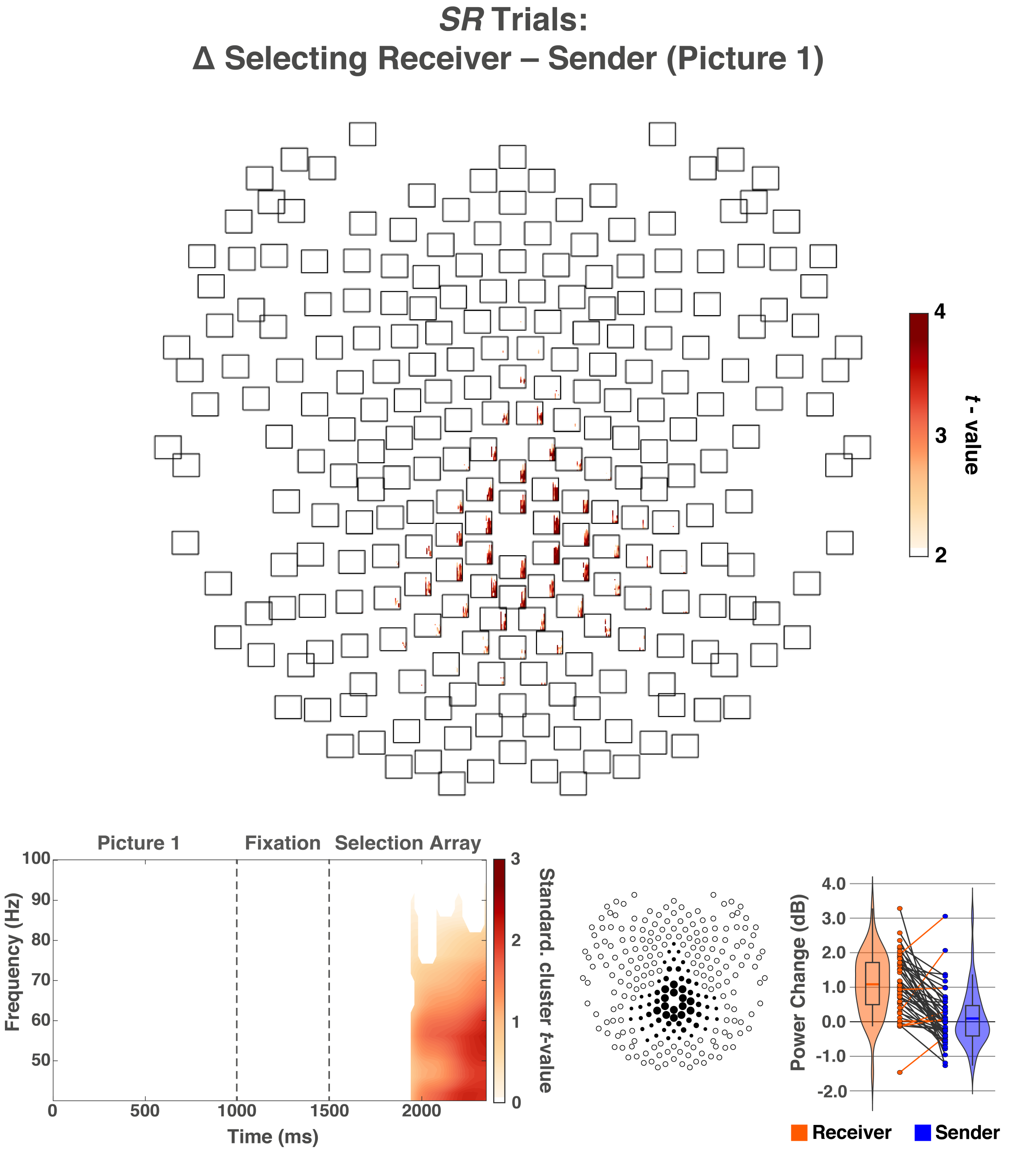

Fig. S13. Illustration of a significant positive cluster (*p = .0002; d = 0.90*) in induced oscillations (40-100 Hz) for *Selecting Receivers* as compared to *Senders* during *SR* trials.

The picture was presented in a time window of 0 to 1000 ms, followed by a fixation cross for 500 ms and the first second of the picture selection array (1500-2500 ms). For a description of the different panels, please refer to Fig. S1 and S5.

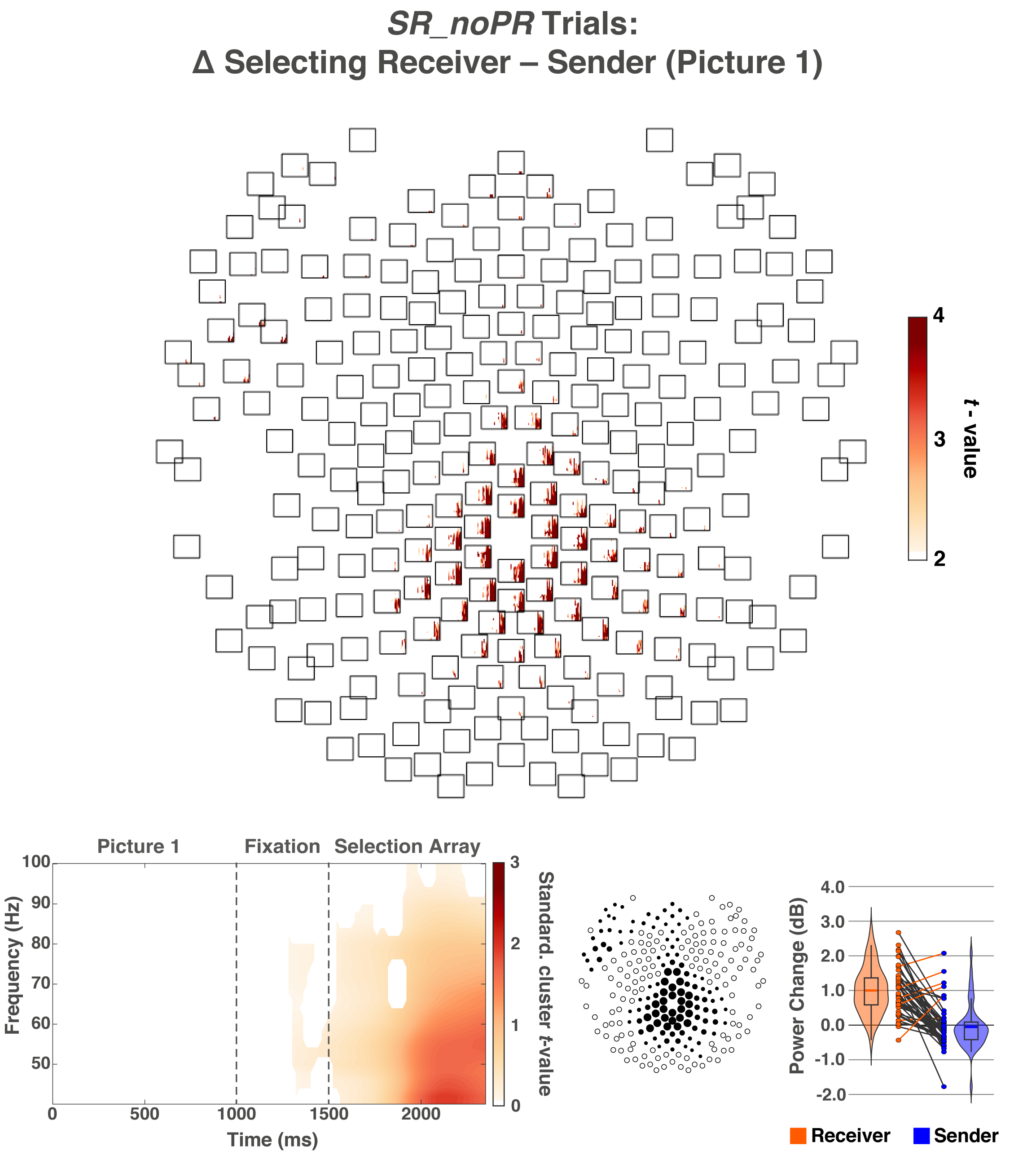

Fig. S14. Illustration of a significant positive cluster (*p = .0002; d = 1.11*) in induced oscillations (40-100 Hz) for *Selecting Receivers* as compared to *Senders* during *SR_noPR* trials.
The picture was presented in a time window of 0 to 1000 ms, followed by a fixation cross for 500 ms and the first second of the picture selection array (1500-2500 ms). For a description of the different panels, please refer to Fig. S1 and S5.
